# RpoN and the Nps and Npa two‐component regulatory system control *pilE* transcription in commensal *Neisseria*

**DOI:** 10.1002/mbo3.713

**Published:** 2018-08-05

**Authors:** María A. Rendón, Beatriz Lona, Mancheong Ma, Magdalene So

**Affiliations:** ^1^ The BIO5 Institute and Department of Immunobiology University of Arizona Tucson Arizona; ^2^ Present address: Sterility Assurance Labs

**Keywords:** *Neisseria*, transcription regulation, two‐component sensors, Type IV fimbriae

## Abstract

Over 20 genes are involved in the biogenesis and function of the *Neisseria* Type IV pilus (Tfp). In the pathogenic species, RpoD and the integration host factor (IHF) protein regulate expression of *pilE*, encoding the Tfp structural subunit. We previously reported that in commensal species, *pilE* transcription is regulated by RpoN, IHF, and activator Npa. Npa has many hallmarks of response regulators in two‐component regulatory systems, leading us to search for its response regulator partner. We report that Npa partners with sensor kinase Nps to control *pilE* transcription. Among the genes involved in Tfp biogenesis and function, only *pilE* is controlled by RpoN and Npa/Nps. We summarize our findings in a model, and discuss the implications of the differential regulation of *pilE* the context of *Neisseria* Tfp biogenesis.

## 1 INTRODUCTION

The Type IV pilus (Tfp) is a multiprotein structure on the surface of many bacteria and archaea (Albers & Jarrell, 2015; Berry & Pelicic, 2015). Tfp of commensal and pathogenic *Neisseria* promote attachment, motility, biofilm formation, horizontal gene transfer, and host cell signaling (Heckels, 1989; Howie, Glogauer, & So, 2005; Ma et al., 2018; Nassif et al., 1994; Pujol, Eugene, Marceau, & Nassif, 1999; Virji et al., 1995). PilE, the name given to the *Neisseria* Tfp structural subunit, is assembled into the pilus filament, and anchored to the membrane by means of a complex machinery (Carbonnelle, Helaine, Nassif, & Pelicic, 2006; Parge et al., 1995).

We reported that transcriptional control of *pilE* differs in pathogenic and commensal species of *Neisseria* (Rendón, Hockenberry, McManus, & So, 2013). In the pathogens, housekeeping sigma factor RpoD initiates *pilE* transcription from the conserved −35 and −10 recognition sequences in the *pilE* promoter (Fyfe, Carrick, & Davies, 1995; Meyer, Billyard, Haas, Storzbach, & So, 1984). In commensals, sigma factor RpoN initiates *pilE* transcription by binding to a highly conserved motif (GGN_10_GC) located −24 and −12 bases upstream of *pilE* (Rendón et al., 2013; Wormann et al., 2016).

RpoN regulation of *pilE* also requires integration host factor (IHF) and an activator, Npa (Rendón et al., 2013). IHF enhances the efficiency of *pilE* transcription in both pathogenic and commensal *Neisseria* species, albeit by different mechanisms (Hill et al., 1997; Rendón et al., 2013). In RpoD‐dependent promoters, such as in *Neisseria gonorrhoeae* (Ngo) *pilE*, IHF is thought to bend the DNA, thereby stabilizing the interaction of the promoter with RpoD and enhancing transcription (Giladi et al., 1998; Hill et al., 1997). IHF appears to play a similar role in the RpoN‐dependent *pilE* promoter of commensal *Neisseria*. If IHF cannot interact with its binding site, transcription of *pilE* decreases significantly (Rendón et al., 2013). Finally, Npa is a positive regulator of *pilE*. Point mutations in its binding site, the upstream activator sequence (UAS), or deletion of *npa* abolished *pilE* expression (Rendón et al., 2013).

Npa has motifs typical of response regulators of two‐component regulatory systems (TCRS), namely, a receiver domain in the N terminus and an output domain in the C terminus (Carrick, Fyfe, & Davies, 2000; Jung, Fried, Behr, & Heermann, 2012). A TCRS consists of a membrane‐bound sensor kinase (SK) and a cytoplasmic response regulator (RR). The SK autophosphorylates upon detection of a signal, and transfers the phosphoryl group to the RR, which then activates or represses gene expression. In the RR, the phosphorylated amino acid is a highly conserved aspartate. We previously speculated that aspartate 58 (D58) in Npa is the phosphorylated residue (Rendón et al., 2013). Since Npa has hallmarks of a response regulator, we searched the genome of commensal *Neisseria elongata* (Nel) for its SK counterpart, and found Open Reading Frame (ORF) (NEIELOOT_00058) whose deduced amino acid sequence has domains typically found in SK. NEIELOOT_00058 is present in all other commensal *Neisseria* genomes published on the web.

In this work, we tested the hypothesis that NEIELOOT_00058 is the SK that activates Npa to regulate transcription of *pilE*, using *Neisseria elongata* (Nel) as the model commensal. We show that Nps is required for *pilE* transcription, and that Nps and Npa act in concert as a TCRS. Finally, we show that RpoN and Nps/Npa control transcription only of *pilE* and not the other Tfp biogenesis genes reported to date. The implication of these different control mechanisms for Type IV pilus biogenesis is discussed.

## 2 EXPERIMENTAL PROCEDURES

### 2.1 Strains, plasmids, and growth conditions

All strains and plasmids used in this study are listed in Table S1. *Neisseria* strains were routinely grown at 37°C with 5% CO_2_ in GCB agar or liquid GCB containing Kellogg's supplements (Kellogg, Peacock, Deacon, Brown, & Pirkle, 1963), unless otherwise indicated. *Escherichia coli* strains were grown at 37°C in LB media (Bertani, 1951). Antibiotics were added when needed at specified concentrations. Chloramphenicol was added at a final concentration of 5 μg/ml, and kanamycin at 50 μg/ml for *Neisseria*. For *E. coli*, chloramphenicol was used at 30 μg/ml and kanamycin at 50 μg/ml.

### 2.2 Bioinformatics

Various online programs were used to analyze the sequences. To predict conserved domains, NCBI domain (http://www.ncbi.nlm.nih.gov/Structure/cdd/wrpsb.cgi/) was used. TMMOD server was used to predict transmembrane domains (Kahsay, Gao, & Liao, 2005) (http://liao.cis.udel.edu/website/servers/TMMOD/scripts/frame.php?p=submit).

Protein sequence alignments were done using Clustal Omega (http://www.ebi.ac.uk/Tools/msa/clustalo/) (Sievers et al., 2011). Protein homology and similarity were determined using the FASTA protein similarity search tool (http://www.ebi.ac.uk/Tools/sss/fasta/) (Pearson, 1990).

### 2.3 Construction of mutants

The *nps* and *npa* ORFs overlap by 11 bases, to ensure deletion of *nps* would not affect *npa*, we designed a construct in which 92% of *nps* was deleted. First, a kanamycin resistance gene from plasmid pHSS6 (Seifert, Chen, So, & Heffron, 1986) was amplified with primers MR327A and MR328A (Table S2). The purified PCR product was transformed into Nel 29315. Transformants were selected in GCB containing 50 mg/ml of kanamycin. Ten kanamycin‐resistant colonies were sequenced to confirm *nps* replacement. One kanamycin‐resistant clone was selected and transformed with a 321 bp piece of DNA of which 117 bases were from *nps*. The transformation mix was serially diluted, and plated onto GCB agar. After 24 hr, individual colonies were selected and grown on GCB without antibiotic, and a replica in GCB‐Km. Km‐sensitive clones were selected and analyzed, first by PCR, and then by sequencing to confirm that the kanamycin resistance gene was removed (Figure S7).

### 2.4 Site directed mutagenesis

A kanamycin resistance gene was cloned between the HincII and HindIII sites in pUC19, to generate plasmid pUC19‐Km. Primers MR313 and MR314 were used to amplify the *nps*‐*npa* locus and the amplicon was inserted between the EcoRI and BamHI sites of pUC19‐Km. The site directed mutagenesis protocol in (Heckman & Pease, 2007) was used to generate mutants Nel *nps* H325A, Nel *npa* D58A, Nel *npa*D58E, Nel Δ*nps‐npa* D58E, and Nel *nps* H325A‐*npa* D58E. All the primers used are listed in Table S2.

### 2.5 Complementation of mutant strains

Complemented strains were constructed by placing the wt gene of interest under control of the inducible *lac* promoter, and inserting the construct into the *proB* site in Nel. For this protocol, we constructed plasmid pML2 (Figure S8) by modifying plasmid pKH37 (Kohler, Hamilton, Cloud‐Hansen, & Dillard, 2007). Briefly, a 650 bp fragment of the 5′‐end of *proB* gene, obtained by PCR using primers MR346 and MR347, was used to replace the *aspC* gene in the FspI and PciI sites generating plasmid pML1. The *lctP* gene was removed by digestion with SphI and KpnI. A 550 pb fragment of the 3′‐end of *proB*, generated using primers MR348a and MR349, was cloned into the SphI and KpnI sites of pML1 creating plasmid pML2. The multiple cloning site, *lacPO*,* lacIq*, and *cat* remained intact.

Nps was amplified using primers MR350 and MR 351 and cloned into the PacI and SacI sites of pML2 generating plasmid pML2‐i*nps*. Npa was amplified using primers MR216a and MR216b Nel and cloned into the PacI and XhoI sites of pML2 generating plasmid pML2‐i*npa*. Nel mutants were transformed with the corresponding plasmid using the colony transformation protocol described in (Dillard, 2011). Transformants were selected in GCB with chloramphenicol (5 μg/ml). Complemented strains were verified by PCR, sequencing, and RT‐PCR.

### 2.6 RNA extraction and RT‐PCR

Total *Neisseria* RNA was extracted using Trizol (Invitrogen) and treated with DNAse free (Ambion) to remove DNA, as recommended by the manufacturer. The integrity of the RNA was determined by agarose gel electrophoresis. cDNA was generated with 1,000 μg of RNA using M‐MLV (Promega) per manufacturer's instructions. Reactions without reverse transcriptase were used as negative controls. To ensure that equal amounts of RNA were used, a reaction to amplify 16S rRNA was performed. All primers used, and gene products are listed in Table S2.

### 2.7 Polyclonal anti‐PilE antibodies

Pili from Nel wt were purified from GCB agar plates as previously described (Rendón et al., 2013). Briefly, Nel was grown for 16 hr on GCB agar. Bacteria were collected in 2 ml of 0.3 M ethanolamine. Pili were sheared off by vortexing the bacteria for 1 min. Pili were precipitated by incubating the solution with 300 μl of saturated ammonium sulfate for 30 min at RT. Precipitated pili were collected by centrifugation (17,000 × *g* for 30 min). Polyclonal antibodies to this pilus preparation were raised by immunization of a rabbit with 4 weekly doses of purified pili (Alpha Diagnostic Intl. Inc). Nonspecific antibodies were removed from the immune sera by absorbing with intact Nel Δ*pilE* cells. Finally, specific anti‐PilE antibodies were captured by affinity purification using purified Nel pilin. The antibodies were released by addition of 0.2 M glycine pH 2.5, following by incubation at RT for 5 min. The solution with antibodies was collected and neutralized with 1 M Tris–HCl pH 8. The specificity of the antibodies was tested by western blot (Figure S9).

### 2.8 Western blot

Equal amounts of bacteria (7 × 10^8^ bacteria) were suspended in sample buffer. Ten microlitre of sample was added to each lane of a 15% acrylamide gel. To ensure a good separation, the gel was run at 90 volts for 2.5 hr. Proteins were transferred onto a nitrocellulose membrane using a Trans‐Blot SD semidry transfer cell (Bio‐Rad). The membrane was dried and then blocked with 5% milk‐TBST for 1 hr. Anti‐PilE_Nel_ antibodies were diluted 1:10,000 (in 5% milk‐TBST) and incubated with the membrane for 1 hr. The membrane was washed 3× and incubated with secondary goat anti‐rabbit antibodies (LICOR) for 1 hr. The western blot was visualized using an Odyssey Clx Infrared Imaging System (LICOR) instrument.

### 2.9 Determination of transcription initiation site

Total RNA was purified as described above, followed by mRNA enrichment using the MICROBExpress bacterial mRNA enrichment kit (Ambion) per the manufacturer's instructions. A 5′ Rapid amplification of cDNA ends (RACE) reaction was used to determine the transcription initiation site (TIS) using the SMARTer® RACE 5′/3′ kit (Takara‐Clontech). Primers used to determine TIS and their targets are described in Table S2. The product obtained in each reaction was cloned into pCR2.1 (Life Technologies) and transformed into *E. coli* DH5. Colonies were selected for sequencing using universal primers M13 forward and M13 reverse.

## 3 RESULTS

### 3.1 *nps* and *npa* form an operon

**Figure 1:**
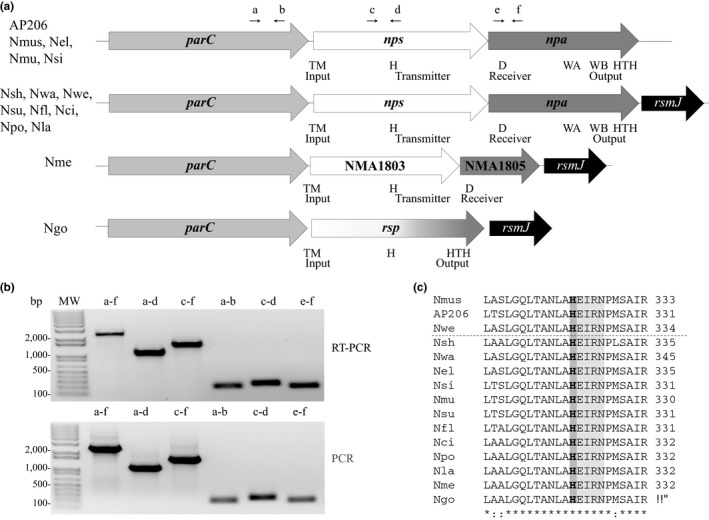
The *nps*‐*npa* operon in *Neisseria* species. (a) The *nps*‐*npa* locus in commensal *Neisseria*, and the analogous locus in pathogens Nme and Ngo. The small black arrows above the genes indicate the binding site of the primers used in the study (b). a, primer MR295, b, primer MR297; c, primer MR299; d, primer MR300; e, primer MR298; f, primer MR296. H, histidine, D, aspartate, WA, walker box A, WB, walker box B, HTH, Helix‐turn‐helix. (b) Top panel: Right three lanes: *parC* to *npa*,* parC*‐*nps*, and *nps*‐*npa* transcripts. Left three lanes: *parC*,* nps* and *npa* transcripts. All transcripts were produced by RT‐PCR using primers shown in (a). Bottom panel: Right three lanes: *parC* to *npa*,* parC*‐*nps*, and *nps*‐*npa* amplicons. Left three lanes: *parC*,* nps* and *npa* amplicons. All amplicons were produced by PCR using primers shown in (a). MW, molecular weight markers. (c) Alignment of the H phosphotransfer domain in Nps from *Neisseria* species. The phosphorylatable histidine is in bold type and boxed in dark gray. The canonical phosphatase motif (ExxN) in *Pseudomonas aeruginosa* PilS that is present in the Nps homolog is boxed in light gray. The dashed line separates Nps in animal and human *Neisseria*. (*) identical residues; (:) highly conserved residues. SAM, S‐adenosyl methyl transferase; Nmus, *N. musculi*; AP206, *Neisseria* sp isolated from rhesus macaque; Nwe, *N. weaveri*; Nsh, *N*. *shayeganii*; Nwa, *N. wadsworthii*; Nel, *N. elongata*; Nmu, *N. mucosa*; Nsi, *N. sicca*; Nsu, *N. subflava*; Nfl, *N. flavescens*; Nci, *N. cinerea*; Npo, *N. polysaccharea*; Nla, *N. lactamica*; Nme, *N. meningitidis;* Ngo, *N. gonorrhoeae*

We reported that *pilE* transcription in commensal *Neisseria* requires RpoN and the activator Npa (Rendón et al., 2013). Several domains characteristic of TCRS response regulators are present in Npa. Upstream of *npa* is ORF (NEIELOOT_00058, accession number: AJE19474.1) (Figure 1a), whose deduced amino acid sequence contains motifs typically found in SKs. We provisionally named this ORF *Neisseria* pilus sensor (Nps). Upstream of *nps* and *npa* is the DNA topoisomerase IV subunit A, *parC* (accession number: AJE19475.1); all three ORFs are arranged in the same orientation. This organization of the three genes is identical in the human commensals *N. mucosa* (Nmu) and *N. sicca* (Nsi), and in the animal commensals *N. musculi* (Nmus) and AP206, a *Neisseria* sp isolated from rhesus macaque (Weyand et al., 2013). In *Neisseria weaverii* (Nwe), *Neisseria shayeganii* (Nsh), *Neisseria wadsworthii* (Nwa), *Neisseria subflava* (Nsu), *Neisseria flavescens* (Nfl), *Neisseria cinerea* (Nci), *Neisseria polysaccharea* (Npo), and *Neisseria lactamic*a (Nla), *npa* is followed by a fourth ORF, *rsmJ*, encoding a putative ribosomal RNA small subunit methyl transferase (locus tag NMA1806 in Nme). In pathogen *Neisseria meningitidis* (Nme), *parC* is followed by NMA1803, NMA1805, then *rsmJ*. NMA1803 is a homolog of Nps that lacks the last 76 bases of the ORF (Figure S1). NMA1805 is a homolog of Npa that lacks the last 1260 bases of the ORF, the region that would have encoded the RpoN‐ and DNA‐binding domains (Rendón et al., 2013). In pathogen *Neisseria gonorrhoeae* (Ngo), *parC* is followed by *rsp*, a fusion of *npa* and *nps* (Carrick et al., 2000; Rendón et al., 2013).

The organization of *parC*,* nps*, and *npa*, and the presence of promoter‐like sequences upstream of *parC* suggest the three ORFs comprise an operon. We determined whether these genes are cotranscribed. Using primers specific for each gene (Table S2) in a RT‐PCR reaction of mRNA from log phase bacteria, we confirmed *parC*,* nps*, and *npa* are part of a large transcript (Figure 1b). In Nme, *parC*, NMA1803 (*nps*), NMA1805 (*npa*), and NMA1806 (*rsmJ*) are transcribed as an operon (Jamet, Rousseau, Monfort, Nassif, & Martin, 2010).

### 3.2 Predicted domains of Nps

The closest Nps homologs are PilS of *Kingella kingae* (Kki) (47.5% identity and 75.5% similarity) and PilS of *Pseudomonas aeruginosa* (Pae) (28.8% identity and 64.3% similarity). In Kki and Pae, PilS is required for transcription of *pilA*, encoding the major subunit of their Tfp (Boyd, Koga, & Lory, 1994; Kehl‐Fie, Porsch, Miller, & St Geme, 2009). The N‐terminus of Nps is predicted to span the membrane, and the C terminus to be located in the cytoplasm (TMMOD server) (Kahsay et al., 2005). The N terminus is highly hydrophobic, and harbors the input domain that contains six transmembrane helices interspersed with short loops (residues 21–189) (Figure S1). Residues 313–376 are predicted to function as a transmitter domain, as it contains two motifs, histidine (H) at position 325 and motif E/DxxN/T, that are commonly found in SKs (Figures 1c and S1) (Huynh, Noriega, & Stewart, 2010; Kilmury & Burrows, 2016). When the input domain of a SK detects a signal, the conserved H autophosphorylates, initiating a signaling cascade that activates transcription of the target gene. In Nps, H325 is predicted to participate in the phosphorylation cascade (Figures 1c and S1). These characteristics of Nps lead us to test the hypothesis that Nps is the SK that regulates *pilE* transcription by activating Npa.

### 3.3 *pilE* transcription requires Nps

**Figure 2:**
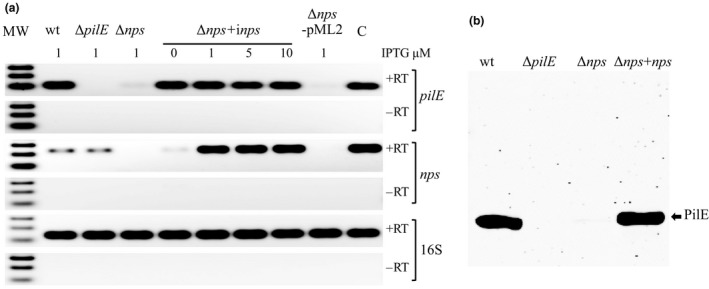
*pilE* mRNA (a) and PilE protein (b) in Nel 29315 wt, Δ*nps* and Δ*npa*. (a) Log phase cells were incubated with varying concentrations of IPTG for 4 hr, at 37°C, and *pilE* or *nps* mRNA from the lysates was measured by RT‐PCR in the presence (+RT) or absence (−RT) of reverse transcriptase, using *nps*‐specific primers (see Table S2). The 16S rRNA reaction was used as a loading control and control for mRNA stability. Nel Δ*pilE* was included as negative control. Lane labeled “C” denotes control for PCR reactions, performed in the presence of DNA (+RT) or H_2_O (−RT). (b) PilE levels were detected by western blot using polyclonal antibodies raised to Nel PilE. Arrow points to PilE. Nel Δ*pilE* was included as negative control. Δ*nps*+i*nps*, complemented strain expressing an IPTG‐inducible wt *nps*; Δ*nps*+pML2, control strain containing a plasmid with the *lacZ* promoter

We created an in‐frame deletion of *nps* in Nel 29315 (see Methods). NelΔ*nps*, failed to produce detectable amounts of *pilE* mRNA (Table 1 and Figure 2). The complemented strain, NelΔ*nps*+i*nps*, expressing *nps* controlled by an Isopropyl β‐D‐1‐thiogalactopyranoside (IPTG)‐inducible promoter, restored *pilE* mRNA production. IPTG‐inducible promoters are known to be leaky, and Nel Δ*nps*+i*nps* produced low levels of mRNA even in the absence of the inducer (Figure 2a). Nel wt and NelΔ*nps*+i*nps* produced similar amounts of PilE, as detected by western blot (Figure 2b). Consistent with the absence of PilE, NelΔ*nps* produced colonies with a non‐piliated phenotype and did not form microcolonies (Figure S2). *npa* mRNA levels were identical in both wt and Δ*nps*, indicating the absence of *pilE* mRNA was due to the absence of *nps*, and not the absence of *npa* (Figure S3b). These results show that *nps* is essential for *pilE* transcription.

**Table 1:** Phenotype of Nel29315 mutants

Strain	*pilE* mRNA	PilE protein	Microcolony formation	Reference
Nel 29315 (wt)	+	+	+	Marri et al. (2010)
Nel 29315 Δ*pilE*	−	−	−	Higashi et al. (2011)
Nel 29315 Δ*nps*	−	−	−	This work
Nel 29315 Δ*nps+*i*nps*	+	+	+	This work
Nel 29315 *nps* H325A	−	−	−	This work
Nel 29315 *nps* H325A+i*nps*	+	+	+	This work
Nel 29315 Δ*npa*	−	−	−	Rendón et al. (2013)
Nel 29315 Δ*npa*+i*npa*	+	+	+	This work
Nel 29315 *npa* D58A	−	−	−	This work
Nel 29315 *npa* D58A+i*npa*	+	+	+	This work
Nel 29315 *npa* D58E	+	+	+	This work
Nel 29315 Δ*nps‐npa* D58E	+	+	+	This work
Nel 29315 *nps* H325A‐*npa* D58E	+	+	−/+	This work

### 3.4 Nps and Npa form a two‐component regulatory system

**Figure 3:**
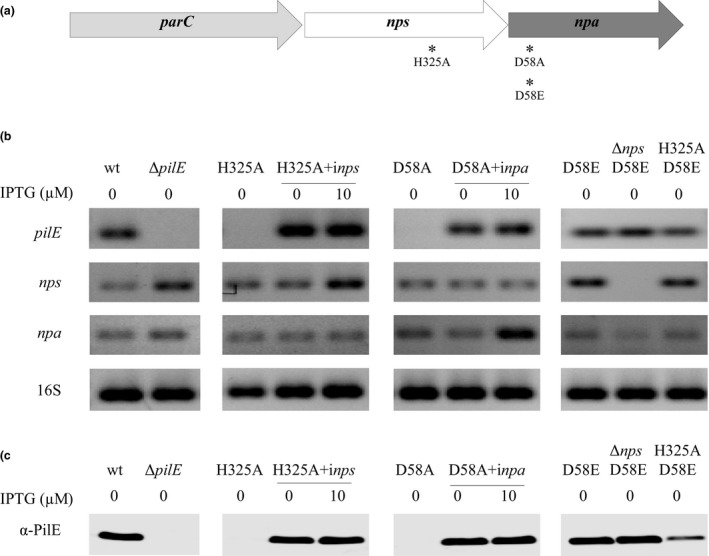
*pilE*,* nps*, and *npa* transcript levels in Nel 29315 wt and *nps* and *npa* mutants. (a) Point mutations in the *nps* and *npa* genes are indicated by an asterisk. (b) Log phase cells were incubated with varying concentrations of IPTG for 4 hr, at 37°C. *pilE*,* nps*,* or npa* mRNA from the lysates was measured by RT‐PCR in the presence (+RT) or absence (−RT) of reverse transcriptase, using gene‐specific primers (see Table S2). The 16S rRNA reaction was used as a loading control and control for mRNA stability. Name of each mutant appears above each column. Primers targeting 16S rRNA were used as loading control and control of mRNA stability. IPTG concentration used to induce the genes for complementation appears above the columns. (c) Detection of PilE by western blot using anti‐PilE antibodies. H325A+inps, *nps* H325 complemented with wt *nps* under control of the IPTG‐inducible promoter; D58A+i*npa*,* npa* D58A complemented with wt *npa* under control of the IPTG‐inducible promoter; H325A D58E, *nps‐npa* double mutant

Upon activation, the SK transfers its phosphoryl group from the conserved H to a conserved aspartate (D) on the RR (Zschiedrich, Keidel, & Szurmant, 2016). We tested the hypothesis that the conserved H325 in Nps and D58 in Npa participate in the phosphorylation cascade, by replacing Nps H325 and Npa D58 with alanine (A) (Figure 3a). *nps*H325A is predicted not to autophosphorylate, and consequently unable to activate Npa and *pilE* transcription. As expected, *pilE* mRNA and PilE levels were undetectable in *nps*H325A and in *npa*D58A (Figure 3b,c). These mutants formed colonies with a non‐piliated morphology and did not form microcolonies (Table 1 and Figure S2). Complementation of *nps* or *npa* with the cognate IPTG‐inducible wt gene (Nel *nps*H325A+i*nps* and *npa*D58A+i*npa*, respectively) restored *pilE* transcription, PilE production and microcolony formation (Table 1, Figures 3b,c and S2). Since these strains carry the mutated copy of *nps* or *npa* in addition to the inducible copy, the amount of IPTG needed to restore *pilE* levels was relatively high (5 μM for Nel *nps*H325 + i*nps* and 10 μM for Nel *npa*D58A+i*npa*). We confirmed by RT‐PCR that levels of *nps* and *npa* transcripts were not affected by the point mutations in Nel *nps*H325A and Nel *npa*D58A (Figure 3b).

Phosphorylation of the response regulator NtrC induces a conformational change (Hwang, Thorgeirsson, Lee, Kustu, & Shin, 1999). To mimic such a change, the activatable aspartate (D) in NtrC was substituted with a glutamate (E) (Klose, Weiss, & Kustu, 1993; Lan & Igo, 1998; Moore, Shiau, & Reitzer, 1993). To confirm that D58 in Npa is the phosphorylated amino acid, we made an analogous mutation and created Nel *npa* D58E. Nel *npa*D58E produced *pilE* mRNA and PilE protein at levels similar to wt (Figure 3b,c) and produced piliated colonies on agar (Figure S4). To further confirm Nel *npa*D58E behaves like a phosphorylated RR, we created double mutants Nel Δ*nps*‐*npa* D58E and Nel *nps* H325A‐*npa* D58E. As expected, Nel Δ*nps*‐*npa* D58E and Nel *nps* H325A‐*npa* D58E retained the ability to transcribe *pilE* (Table 1, Figure 3b,c). However, Nel *nps* H325A‐*npa* D58E produced lower amounts of *pilE* mRNA and PilE than the wt parental strain (Figure 3c). The reduction in *pilE* expression in these double mutants was not caused by a defect in *nps* or *npa* expression, since these mutant genes were transcribed at the same level as their wt counterpart.

In sum, the data demonstrate that Nps and Npa work in concert as a TCRS to control *pilE* expression.

### 3.5 Tfp genes other than *pilE* are not controlled by the RpoN/Nps‐Npa regulatory system

In pathogenic *Neisseria*, assembly and function of the Tfp is a complex process that involves over 20 proteins (Carbonnelle et al., 2006). Tfp biogenesis genes are present in all commensal *Neisseria* species (Marri et al., 2010), but how these commensal genes are regulated is unknown. We determined whether RpoN/Nps/Npa regulate expression of these genes in addition to *pilE*.

**Figure 4:**
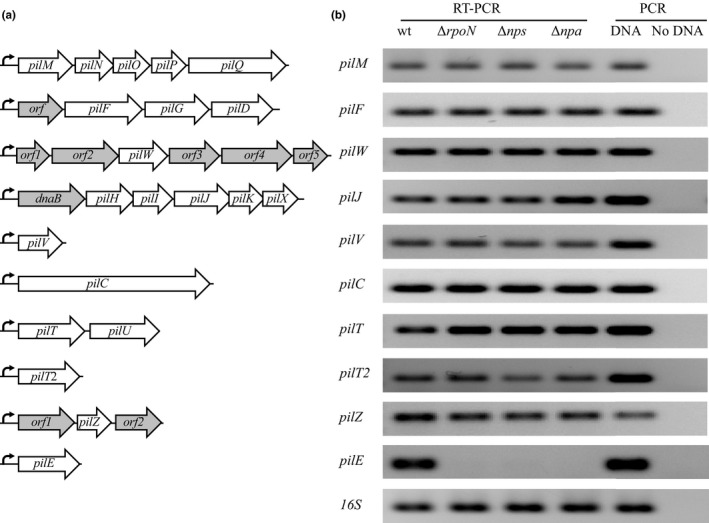
Role of RpoN and Nps/Npa in regulating Tfp biogenesis and function genes. (a) Organization of Tfp biogenesis and function genes in Nel29315. Genes known to play a role in Tfp biogenesis or function are represented by white arrows. Other genes are represented by gray arrows. Dark curved arrow at left indicates the transcriptional initiation site of the mRNA produced at each locus. (b) *pilM*,* pilF*,* pilW*,* pilJ*,* pilV*,* pilC*,* pilT*,* pilT2*, and *pilZ* mRNA was determined in log phase wt, Δ*rpoN*, Δ*nps*, and Δ*npa* cells by RT‐PCR. *pilE* mRNA served as a positive control for *rpoN*,* nps*, and *npa* mutants. 16S rRNA served as the loading control and control of mRNA stability. A PCR reaction in the presence of DNA or in H_2_O (No DNA) served as the control for the amplification reaction

The Tfp genes in Nel 29315 are clustered in ten regions (Figure 4a). Each cluster is transcribed as a polycistronic unit, as determined by RT‐PCR using a combination of primers (Table S2 and Figure S5). One operon contains *pilM*,* pilN*,* pilO*,* pilP*, and *pilQ* (Figures 4a and S5a). A second operon contains ORF NEIELOOT_00092, followed by *pilF*,* pilG*, and *pilD* (Figures 4a and S5b). The genes in this locus are organized differently in pathogenic *Neisseria*, where *pilF* is convergently transcribed to the operon formed by *pilG*‐*pilD* (Freitag, Seifert, & Koomey, 1995; Lin, Ryan, & Davies, 2011; Tonjum, Freitag, Namork, & Koomey, 1995). A third operon contains six genes (Figures 4a and S5c) only one of which, *pilW*, is known to function in Tfp biogenesis. A fourth operon contains most of the minor pilin genes (*pilH*,* pilI*,* pilJ*,* pilK*, and *pilX*). Another minor pilin gene, *pilV*, is transcribed as a monocistronic unit (Figures 4a and S5d). Nel has only one copy of *pilC*, unlike Nme and Ngo (Marri et al., 2010), and its transcript is also monocistronic (Figure 4a). Like Ngo and Nme, Nel *pilT* and *pilU* are transcribed as one unit (Figures 4a and S5e). In Ngo and Nme, *pilT2* is in a putative operon with *pilZ* (Brown, Helaine, Carbonnelle, & Pelicic, 2010), but in Nel, *pilT*2 is in a separate locus (Figure 4a). Finally, *pilZ* is flanked by ORFs 1 and 2 (NEIELOOT_01544 and NEIELOOT_01542) and the three genes form an operon (Figures 4a and S5f).

To determine whether RpoN and Nps‐Npa control transcription of these nine gene clusters, we selected a gene from each operon and examined its expression in Δ*rpoN*, Δ*nps*, and Δ*npa* mutants. RT‐PCR was performed on mRNA from mid‐log bacteria using specific primers (Table S2). 16s rRNA was use as the loading control and the control for mRNA integrity. A reaction on DNA was conducted as a control for primers specificity. In contrast to *pilE*, transcription of the tested genes was not altered in the Δ*rpoN*, Δ*nps*, and Δ*npa* mutants when compared to the wt (Figure 4b). The amplicons were not a product of DNA contamination, as judged by lack of a product when RT was excluded from the reaction (data not shown). From these results, we conclude that RpoN and the TCRS Nps‐Npa do not control transcription of these Tfp biogenesis genes other than *pilE*.

Finally, we mapped the TIS of 4 Tfp loci by 5′ RACE (Rendón et al., 2013). In all cases, the TIS mapped to RpoD recognition sequences (Table S3). This confirms that a sigma factor of the RpoD‐family, and not RpoN, regulates expression of these loci.

In conclusion, of all the Tfp biogenesis and function genes described to date, only *pilE* is regulated by RpoN and the TCRS Nps‐Npa.

## 4 DISCUSSION

The genes required for Tfp biogenesis and function are present in animal‐ and human‐dwelling *Neisseria* species (Marri et al., 2010; Weyand et al., 2013, 2016). Tfp gene expression has been studied mostly in the two pathogenic species. In the pathogens, RpoD regulates transcription of *pilE*,* pilT/U*, and *pilC* (Eriksson et al., 2015; Meyer et al., 1984; Taha, Giorgini, & Nassif, 1996). We previously reported that in commensal species of *Neisseria*, RpoN, IHF and the activator Npa regulate *pilE* transcription (Rendón et al., 2013).

Using *Neisseria elongata* as a model commensal, we showed that Nps and Npa act as the SK and response regulator, respectively, of a TCRS that regulates *pilE* transcription (Figures 2 and 3), and that Nps H325 and Npa D58 participate in the phosphorylation events. *nps* H325A and *npa* D58A failed to transcribe *pilE*, while the complemented strains had this activity restored. A D58E substitution, which mimics an activated residue, resulted in constitutive expression of *pilE*. A similar mutation in NtrC altered the conformation of the response regulator, resulting in a constitutively active enzyme (Hwang et al., 1999; Klose et al., 1993; Lan & Igo, 1998; Moore et al., 1993). The *npa* D58E mutation overcomes the need of *nps*, as the double mutants Δ*nps*+*npa* D58E and *nps* H325A+*npa*D58E are able to express *pilE* (Figure 4b). That the double mutant H325A+*npa*D58E produced significantly lower levels of *pilE* transcript than the wt is surprising. At the moment we cannot explain this observation. We know that this is not due to reduced expression of *nps* or *npa* since expression of these genes was not affected (Figure 3b) and no mutation has occurred in the *pilE* locus. In the Δ*pilE* strain used as control, *nps* and *npa* transcripts appear to be slightly increased compared to the wt strain. It is unclear why this occurred. Further studies will determine whether this difference is due to a feedback mechanism in which the absence of *pilE* affects transcription of *nps*/*npa*.

Although the signal‐sensing domains of some TCRS response regulators have been characterized (Hulko et al., 2006; Taylor & Zhulin, 1999; Xu & West, 1999), the signals detected by these regulators are unknown. *In silico* analysis indicates Nps lacks PAS (Per‐Arnt‐Sim), HAMP (domain present in histidine kinases, adenylate cyclases, methyl accepting proteins and phosphatases), and HPT (histidine containing phosphotransfer) motifs. Nps has six membrane‐spanning regions interspersed with very short loops. Pae PilS, the Nps homolog, also has six such transmembrane domains. Pae PilS is both a kinase and phosphatase; it senses the levels of membrane‐bound pilin (PilA) and regulates *pilA* expression through its phosphatase activity (Boyd et al., 1994; Kilmury & Burrows, 2016). The PilS kinase and phosphatase motifs are highly conserved across all *Neisseria* Nps (Figures 1 and S1). The amino acids arginine 24 in PilS, and glutamate 5 and proline 22 in PilA are important for PilS/PilA interactions and PilS autoregulation (Kilmury & Burrows, 2016). These residues are also present in all *Neisseria* Nps and PilE (Figures S1 and S6, respectively). This suggests Nps may, like PilS, sense and respond to membrane‐bound PilE levels.

We attempted unsuccessfully to determine whether Nel Nps detects pH, oxygen, and growth phase (data not shown). Iron depletion affected *pilE* mRNA levels, but from the data we cannot definitely conclude that iron is the signal detected by Nps. Identifying the signal that Nps recognizes will allow us to understand how commensal *Neisseria* adapts to different environmental conditions.

**Figure 5:**
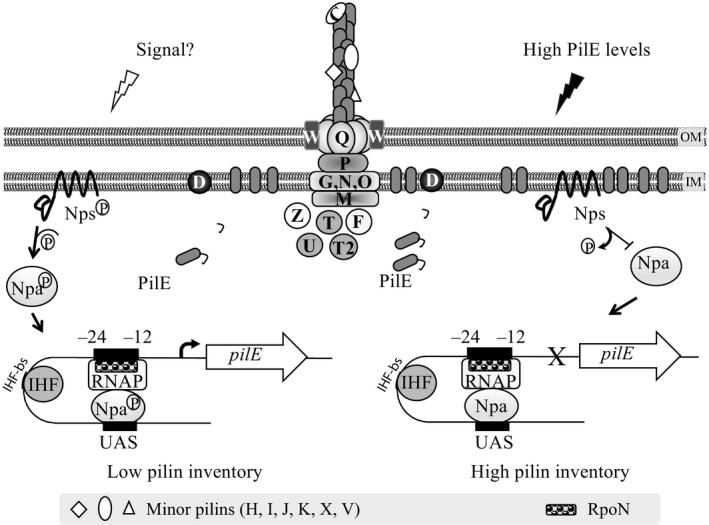
Regulation of *pilE* transcription by RpoN and Nps/Npa in commensal *Neisseria*. Left panel. Upon detection of the signal, Nps autophosphorylates (Nps^P^) and transfers the phosphoryl group to Npa (Npa^P^). Npa^P^ binds the upstream activator sequence (UAS) and activates RpoN, resulting in *pilE* transcription. Transcription is assisted by the binding of integration host factor (IHF) to its binding site (IHF‐bs), bending the DNA and bringing the Npa^P^‐ and RNAP‐DNA complexes into proximity with each other. Right panel. Accumulation of PilE in the inner membrane is detected by Nps. Nps dephosphorylates Npa preventing it from activating RpoN blocking *pilE* transcription

Our model for *pilE* expression in commensal *Neisseria* is summarized in Figure 5. Upon detecting a signal (left), Nps autophosphorylates and transfers the phosphoryl group to Npa (this work). Phosphorylated Npa binds the UAS, promoting RpoN activation of *pilE* transcription (Rendón et al., 2013). *pilE* transcription also requires IHF binding to its cognate sequence, which is situated between the RpoN and Npa recognition sites (Rendón et al., 2013). We hypothesize that this interaction of IHF with DNA bends the DNA, bringing Npa in close proximity to RpoN. In optimal conditions, when the Tfp components necessary for biogenesis are present, PilE would be assembled quickly into pili. If PilE accumulates in the membrane (Figure 5, right panel), Nps would sense this, dephosphorylate Npa, and prevent it from activating RpoN. As a result, *pilE* transcription is abolished.

Our model implies that Tfp biogenesis genes other than *pilE* are constitutively expressed. Our findings indicate they are transcribed from a RpoD‐dependent promoter (Figure 4), but they do not allow us to draw a conclusion about whether they are under more stringent regulation—for instance by an enhancer or a repressor. Future studies are needed to determine whether these genes are controlled by an intricate regulatory system(s).

Why have commensal and pathogenic *Neisseria* evolved different mechanisms to regulate *pilE*? This question might be explained by current information on *Neisseria* Tfp biogenesis. A large number of proteins situated in the inner membrane, periplasm and outer membrane are involved in the assembly and export of PilE and minor pilins (Brown et al., 2010; Carbonnelle et al., 2006; Winther‐Larsen et al., 2005; Wolfgang, van Putten, Hayes, Dorward, & Koomey, 2000). The PilF ATPase incorporates PilE subunits into the Tfp fiber (Freitag et al., 1995) and the PilT ATPase causes the Tfp fiber to retract, presumably by disassembling these subunits from the fiber base (Wolfgang, Lauer, et al., 1998). If the Tfp assembly/disassembly machinery malfunctions, PilE accumulates in the inner membrane and becomes toxic to the bacterium (Carbonnelle et al., 2006; Wolfgang et al., 2000). It is not unreasonable to speculate that the RpoN/Nps/Npa system serves not only to aid the bacterium to adapt to new niches but also to relieve the toxicity of membrane‐bound PilE.

In the pathogens, *pilE* is expressed constitutively (Fyfe et al., 1995; Laskos, Dillard, Seifert, Fyfe, & Davies, 1998; Meyer et al., 1984). Excess PilE is secreted as soluble truncated pilins (S pilin) (Jonsson, Pfeifer, & Normark, 1992; Koomey, Bergstrom, Blake, & Swanson, 1991), and this process is proposed to relieve the toxicity of membrane‐bound PilE (Winther‐Larsen et al., 2005; Wolfgang, Park, Hayes, van Putten, & Koomey, 1998; Wolfgang et al., 2000). In addition, Ngo regulates *pilE* transcription under certain conditions. *pilE* expression is decreased in a *pilF* mutant and increased in a *pilT* mutant (Dietrich, Mollenkopf, So, & Friedrich, 2009), by unknown mechanism(s). Ngo encodes a modified version of the Nps/Npa TCRS, termed Rsp; however, Rsp does not appear to play a role in *pilE* transcription (Carrick et al., 2000). A number of candidates have been proposed to regulate *pilE* (De Reuse & Taha, 1997; Deghmane, Giorgini, Larribe, Alonso, & Taha, 2002); however, none of these has been proven conclusively to control *pilE* transcription in Ngo or Nme. Future studies will allow a better understanding of *pilE* transcriptional in the pathogens.

## CONFLICT OF INTEREST

The authors declare not conflict of interest.

## DATA ACCESSIBILITY

The authors adhere to sharing data and materials policies described in the guidelines for authors.
