# Supplementary material for: RpoN and the Nps and Npa two‐component regulatory system control pilE transcription in commensal Neisseria
Source: Microbiologyopen. 2018 Aug 5;8(5):e00713. doi: 10.1002/mbo3.713 (PMC6528607; doi:10.1002/mbo3.713)
Supplement: Supplementary file 1 [file MBO3-8-e00713-s001.docx]

†

Nmus --MSVNRLFRTHDRETLNERIPGLINIA**R**IAIVLSLLVFHLVSV--YSGNE-TK---NLF 52

AP206 --MS---KSGFQELGNLSERIPGLINIA**R**IAIVLPLLVMHAFGV--YSSGAHVG---MSF 50

Nwe --MSRDKAVLYKNWDGQGEKISGLVNIA**R**IAIVLSLLVFQGLGN--YADGGFVS---TAF 53

Nsh ---MNATAPTQEDWDRQLERLPGLMNIA**R**VSLLLSLVTFHVLVST-YGDEIGLKLRALPV 56

Nwa MSYAKAQLYSRRDWEQQMGRLPVLINLA**R**VTIIISLLVFQIGSDYLYMDTGKMG---SLF 57

Nel --MDKSFFPKTDEWNEQVDRIPWLLNVC**R**LTILLSMFVLYMMSV--FSENAGLK---KMV 53

Nsi --MS---KSGFQELGNLSERIPGLINIA**R**IAIVLPLLVMHAFGV--YSNGTHIG---MSF 50

Nmu --MS---KSGFQELGNLSERIPGLINIA**R**IAIVLPLLVMHAFGV--YSNGTHIG---MSF 50

Nsu --MN---RANLQELGNLRERIPGVINIA**R**IAIVLPLLVLHAFGS--YTGGNLIG---VSL 50

Nfl --MN---RANLQEVGNLRERIPGVINIA**R**IAILLPLLVLHAFGS--YMGGNLIG---VSL 50

Nci -----MVIYNTRELEKLKDRIPNLINIA**R**IAIAFPLLIMHIFGLEVSG-HENLH---TTW 51

Npo -----MVISNPRELEKLKDRIPNLINII**R**VAIVFPLMIMHILGLETGS-RANLH---ASW 51

Nla -----MVISNPRKLEKLKDRIPNLINII**R**VAIVFPLMIMHILGLETGS-RANLH---ASW 51

Nme -----MVISNTRELEKLKDRIPNLINIA**R**IAIVFPLLIMHIFGLEVSG-HENLH---ASW 51

Pae -----MRAERLRLSEEQGQRILRLYHLY**R**LTIGLVLVLLISSELE----DQVLKL--VHP 49

Kki --MQQTAERRHHNWEEQIHRMIGLINIA**R**IAILFSLLIFIAVVSNLVGIAGNQSYLPLIT 58

:: : :: *::: . :. :

Nmus PPLEFYTWAAVYAALILLTIFKPDWQLQSL-DLPNASAVVDISMMLMLTFITGGIDSGFG 111

AP206 PPVEFYSWAALYSFLILLSVARPDWQWQTL-DLPNTSAVVDISMMMILVYIAGGVDSGFG 109

Nwe FSFEFYLWLSAYAAIIIVSILNPTWQSREN-DLPSAWVVVDITMIMWLVYIAGGINSGLG 112

Nsh FSAPFYLWAVVYGTLILISLFAPNWQRQGT-QMPNARAVADITMIAWLMHIGGGVGSGLG 115

Nwa KPVEFYTWAAIYGGIVILTLFKPEWQRQNM-TLPNAAAVADISMIMLLVYMTGGIGMGFG 116

Nel PPTIFYIWAAVYAGIALISIIRPHWVRQENDKLPNASAVVDIVMIMVLVYLTGGIGTGVG 113

Nsi PPVEFYSWAALYSFLILLSVARPDWQWQTL-DLPNASAVVDISMMMILVYIAGGVDSGFG 109

Nmu PPVEFYSWAALYSFLILLSVARPDWQWQTL-DLPNASAVVDISM-MILVYIAGGVDSGFG 108

Nsu PAVEFYIWVTLYFFLIMLSVLRPGWQWQSL-DLPNASAVVDITMMMVLVYISGGTASGFG 109

Nfl PDVGFYIWVTLYFFLIMLSVFRPGWQWQSL-DLPNAGAVVDITMMMVLVYISGGTASGFG 109

Nci TTWAFYLWLAIACWMIFFSILNPHWQWQAL-RIPSFSAVADITLIGILTYLFGGIDSGFG 110

Npo TAWAFYVWLAIACWLIFFSIIHPHWQWQSL-KMPRFSAVADITMIGVLTYLFGGIDSGFG 110

Nla TAWAFYVWLAIACWLIFFSIIHPHWQWQSL-KMPRFSAVADITMIGVLTYLFGGIDSGFG 110

Nme TAWAFYVWLAISCWLIFFSIIHPHWQWQSL-KMPRFSAVADITMIGVLTYLFGGIDSGFG 110

Pae ELFHVGSWCYLVFNILVALFLPPSRQLLPI----FILALTDVLMLCGLFYAGGGVPSGIG 105

Kki NMAWIKIWCIAYSALIGVSLFRPEWQLQGQNKLPNISSVIDISMIAALVFLAGGVESGFA 118

. * : . * : *: : * . ** *..

Nmus ILVLPFIATSCLLSHGRYPMLYASYAFLLILLSMFLSDQITFPPLE--W---DSRSIVSA 166

AP206 ILVLPFVATSCLLSYGHYPMLYAGYASMLFFFNLLLDGSIRLHPFD--W---DTQPLITT 164

Nwe VLVLPFVVTSCLISAGRYPLLYGSYTTMLLLVNLLANGDMQGSPAS--W---NVTAVVMA 167

Nsh ILILPFLVTSCLMSYGRYSLLYASYAVMLLLL----STSMLYWPFK---DDSDASFMVQT 168

Nwa ALVLPFVATSCLLSYGRYPMLYASYATLLIIFCLLLSDHMIFNIIDKKWNGTDFRPFIIG 176

Nel ILVLPFVATSCMLSYGRFPALYAGFTTVCILCVMFLSDQLSLDADT--WDG---RNIGTA 168

Nsi ILVLPFVATSCLLSYGHYPMLYAGYASMLFFFNLLLDGSIRLHPFD--W---DTQPLITT 164

Nmu ILVLPFVATSCLLSYGHYPMLYAGYASMLFFFNLLLDGSIRLHPFD--W---DTQPLITT 163

Nsu ILVLPFVATSCLLSYGHYPMLYAGYTAMLFILNLFLDGSMRFDSFN--W---DAKSVANS 164

Nfl ILVLPFVATSCLLSYGHYPMLYAGYTAMLFILNLFLDGSMRFDSFN--W---DAKSMLNS 164

Nci ILILPFVVSSCLLSYGHYPLLYASYASILLILNALADSNINMYPLI-----LDAKTIAHT 165

Npo ILILPFVVCSCLLSYGRYPLLYSSYAAILLIFNAIADGNIGKYPLI-----SDARTASAT 165

Nla ILILPFVVCSCLLSYGRYPLLYSSYAAILLIFNAIADGDIGKYPLI-----SDARTASAT 165

Nme ILILPFVVCSCLLSYGRYPLLYASYAAILLIFNAIADGDIGKYPHI-----SDARTASAT 165

Pae SLLVVAVAIANILLRGRIGLVIAAAASLGLLYLTFFLSLSSPD---------ATNHYVQA 156

Kki ILVLPFLATSCLLSYGRFPLLYGSYVALLVSFDVFWHLQ----PFSSFVVRDNLNLLTSQ 174

*:: :. : :: *: : .. . : .

Nmus ALLAGACYLVAALTAFSATYLQAATESAEKHQLAYRRVSGLNRLVLNRVQEAVIVIDASQ 226

AP206 VLLSGACYLVAMLTSFAARYLEQATESASRHQLAYRRISGLNRLVLNRVQEAVIVIDSTQ 224

Nwe AFLSGASFLVAALTAFLASYLQEATESANENQQAYRRVSGLNRLVLNRVQEAVVVLDTEQ 227

Nsh VVLSGSCYLVAVLTSFSASFLNKATSSLSRHRRAFDRLKGLNELVLNHVNEAVVVLDVGQ 228

Nwa LFLIGASYLVAGLTSLSVNRLKAATDSADKHKQAFNRVSGLNKLVLNRVQEAVVVLDMDQ 236

Nel IMLIGASFAVAYLTSYSATFLRDATASARKHKRNYNRVRGLNQLVLNRVQEAVIVIDPEL 228

Nsi VLLCGACYLVAMLTSFAARYLEQATESASRHQLAYRRISGLNRLVLNRVQEAVIVIDSTQ 224

Nmu VLLSGACYLVAMLTSFAARYLEQATESASRHQLAYRRISGLNRLVLNRVQEAVIVIDSTQ 223

Nsu LMLIGASYLVAMLTSFAARYLEQATESASRHQLAYRRISGLNHLVLNRVQEAVVVIDATQ 224

Nfl LMLIGAGYLVAMLTSFAAHYLEQATESASRHQLAYRRISGLNRLVLNRVQEAVVVIDATQ 224

Nci FILVAGSYFVAMIASLSVRYIDRAGKLAHENHVAYRRIKGLNQIVLNRVQEAVVVINVEH 225

Npo FILVAASYLSAIFTSLSVRYIDSAGQLARDNHLAYRRIKGLSQTVLERVQEAVVVINTEG 225

Nla FILVAASYLSAIFTSLSVRYIDSASQLARDNHLAYRRIKGLSQTVLERVQEAVVVINTEG 225

Nme FILVAASYLSAIFTSLSVRYIDSAGQLARDNHLAYRRIKGLSQTVLERVQEAVVVINTEG 225

Pae GGLGTLCFAAALVIQALVRRQEQTETLAEERAETVANLEELNALILQRMRTGILVVDSRQ 216

Kki IVLIAACYLVPLLTSFSAEYLASADARILRHKTAFERMSGLNHIVLNRVQEAVIVLDAQQ 234

* : . . : .: *. :*:::. .::*::

Nmus RVWLFNRQAKTY-----FPGLQADKQETVFAELVSRWQYQPDKNFETD-IHIFQHSMHVR 280

AP206 RVWLFNKQAKTY-----FPGLVVDQQEVVFGELVTRWQYHPDKPFETD-IHIFQHAMHVR 278

Nwe TVWLFNRQAKTY-----FPDLEIDRQHNVFGELIEQWQQQPDRVFEID-IHIYQHAMHVR 281

Nsh RVWLMNRQAQNY-----FPWLQREQQAPAFAWLVQRWQRYPSRAFVTR-TEIDGQAVQIR 282

Nwa RIWLFNAQAKTY-----FPSLAIDNQETIFSDLIRQWHHAPEKSFETD-IHLHQHSMRVR 290

Nel KVWLFNRQAKNY-----FSGLAAEHKEPIFEDLVARWLLNPEKSFEAD-IHLHRFSMHVR 282

Nsi RVWLFNKQAKTY-----FPGLVVDQQEVVFGELVTRWQYHPDKPFETD-IHIFQHAMHVR 278

Nmu RVWLFNKQAKTY-----FPGLVVDQQEVVFGELVTRWQYHPDKPFETD-IHIFQHAMHVR 277

Nsu RVWLFNKQAKIY-----FPSLIIDQQEIVFGELVARWQRQPDKPFETD-IHIFQHAMHVR 278

Nfl RVWLFNKQAKIY-----FPSLIIDQQEIVFGELVARWQRQPDKPFETD-IHIFQHAMHVR 278

Nci QTILFNKKAKDL-----LPMLEIGQHTALFDPVAVLWDKTSSRTFERH-IDTPELTARVR 279

Npo LAVLFNRKAKDL-----FPALEIGRRAGLSDSAAELWDQAAPHTFEYV-LGTPGLNAGIR 279

Nla LAVLFNRKAKDL-----FPALEIGRRADLSDSAAELWDQASPHTFEYV-LGTPGLNAGIR 279

Nme LAVLFNRKAKDL-----FPALEIGRRADLSDSAAELWDQASPHTFEYV-LGTLGLTARIR 279

Pae AILLANQAALGLLRQDDVQGASLGRHSPMLMHCMKQWRLNPSLRPPTLKVVPDGPTVQPS 276

Kki QVWLFNDQAQQY-----IPYLTVGKTVEPFGSIIQKWRKQPARIFEDD-CVLMYQPMHVR 288

* * * . . *

Nmus AVPLIQ--EKTELLMLFVRSLREVAAEALATKLASLGQLTANLA**H**EIRNPMSAIRHASDL 338

AP206 AVPLIQ--EQTELLMLYVRSLREVAAEAMSTKLTSLGQLTANLA**H**EIRNPMSAIRHASDL 336

Nwe AVPLIQ--EDTELLMLYIRSLREVAAEVMSTKLASLGQLTANLA**H**EIRNPMSAIRHANDL 339

Nsh ARPLVR--EDESLLMLFIRSQKDLAAEALSTKLAALGQLTANLA**H**EIRNPLSAIRQSNGL 340

Nwa AVPLVQSDEKTELLMLFIRSLREVAAEAMATKLASLGQLTANLA**H**EIRNPMSAIRHANDL 350

Nel AVPMVQ--EDGKLLMLFLRSLREIAAEAMATKLASLGQLTANLA**H**EIRNPMSAIRHANDL 340

Nsi AVPLIQ--EQTELLMLYVRSLREVAAEAMSTKLTSLGQLTANLA**H**EIRNPMSAIRHASDL 336

Nmu AVPLIQ--EQTELLMLYVRSLREVAAEAMSTKLTSLGQLTANLA**H**EIRNPMSAIRHASDL 335

Nsu AVPLIQ--EQTELLMLYVRSLREVAAEAMSTKLTSLGQLTANLA**H**EIRNPMSAIRHASDL 336

Nfl AVPLIQ--EQTELLMLYVRSLREVAAEAMSTKLTALGQLTANLA**H**EIRNPMSAIRHASDL 336

Nci AIPMNK--EQNNLLVLYIRPQNEIQSEALSVKLAALGQLTANLA**H**EIRNPMSAIRHANDL 337

Npo AVPVNK--GPDKLLILYIRPQSEIQAEALSVKLAALGQLTANLA**H**EIRNPMSAIRHANDL 337

Nla AVPVNK--EPDKLLILYIRPQSEIQAEALSVKLAALGQLTANLA**H**EIRNPMSAIRHANDL 337

Nme AVPMNK--EPDKLLILYIRPQSEIQAEALSVKLAALGQLTANLA**H**EIRNPMSAIRHANDL 337

Pae FISLNR--EDDQHVLIFLEDISQIAQQAQQMKLAGLGRLTAGIA**H**EIRNPLGAISHAAQL 334

Kki AVPLVQ--ENTKLLMLFIRSEQERQKEAQTVKLTSLGLLTSNLA**H**EIRNPLSAMRQANDL 346

: : . :::::. : :. **:.** **:.:*******:.*: :: *

Nmus LHDEG---GTDATKAKLHSIIDTNIQRIDKMLEDVSLINKRDSVSREPVNLMKFWLAFKQ 395

AP206 LQESDDDAEPDPVKAKLCGIIDSNIQRIDKMLEDISLLNKRDNISREPINLMKFWLDFKQ 396

Nwe LQEDD----MDPTKVKLHNIIDSNIGRIDKMLEDISLINKRDSLSKETVNIMKFWLAFKQ 395

Nsh LSEDN----DNPLTAKLHGIIDNNIARIDKMLEEVSSLNKSDRLNPETINLMAFWLAFKN 396

Nwa LQESM----QDPTNAKLHDIIDSNIRRIDKMLEDVTSLNKKDNISREKINLMKFWLAFKQ 406

Nel LQENI----EDPVSKKLHGIIDGNIRRIDKMLEDISTLNKKDNLGRESINLMKFWLAFKQ 396

Nsi LQESDDDAEPDPVKAKLCGIIDSNIQRIDKMLEDISLLNKRDNISREPINLMKFWLDFKQ 396

Nmu LQESDDDAEPDPVKAKLCGIIDSNIQRIDKMLEDISLLNKRDNISREPINLMKFWLDFKQ 395

Nsu LQEGD---EADPLKAKLYNIIDSNIQRIDKMLEDVSLLNKRDNISRQPINLMKFWLEFKQ 393

Nfl LQEGD---EAAPLKARLYNIIDSNIQRIDKMLEDVSLLNKRDNISRQPINLMKFWLEFKQ 393

Nci LRENAEEEETDPFKVKLCEIIDGNVRRIDKMLEDISSLNKSNKTEREAIDLMQFWIGFKQ 397

Npo LRENMEAGAADPFNAKLCKIIDGNVCRIDKMLEDISSLNKRNKTERETIDLIPFWEEFKQ 397

Nla LRENMEAGAADPFNAKLCKIIDGNVCRIDKMLEDISSLNKRNKTKRETIGLIPFWEEFKQ 397

Nme LRENMEAGAADPFNAKLCKIIDGNVCRIDKMLEDISSLNKRNKTERETIGLIPFWEEFKQ 397

Pae LQESEE---LDAPDRRLTQIIQDQSKRMNLVIENVLQLSRRRQAEPQQLDLKEWLQRFVD 391

Kki LKENNEN--NLPLVARLTNIIEKNIARVDKMIEDVSTLNKRDRLNPQTIDLHKFWFGFMQ 404

* : :* **: : *:: ::*:: :.: : :.: : * :

Nmus EFTLNNPDAVGCLRMNME--GSSLSVLADPMHLQQIMWNLCNNAWRHSRQDQHAITVLIK 453

AP206 EFTLNNSEAIGCLRMNMD--GNNLTVLADVMHIQQIMWNLCNNAWRHSRQDENAITVLIR 454

Nwe EFILNNPAASGCIHLNME--RSNLSVAVDSMHLQQIMWNLCNNAWRHSKKDAQAIMIAVK 453

Nsh EFLLIRPQAAPCIRFHMESGGSPVKVRFDSAHLQQILWNLLNNAWQHGSQQKGSITVLVK 456

Nwa EFTLNNPDAIGSVRMTME--GNNLAVVADSMHLQQIMWNLCNNAWRHSRKDNQAITVLMR 464

Nel EFTLNNPSSVGCIKMKMQ--GKNLTVTADPMHVQQIMWNLCNNAWRHSTKGSDAIQVSIK 454

Nsi EFTLNNSEAIGCLRMNMD--GNNLTVLADVMHIQQIMWNLCNNAWRHSRQDENAITVLIR 454

Nmu EFTLNNSEAIGCLRMNMD--GNNLTVLADVMHIQQIMWNLCNNAWRHSRQDENAITVLIR 453

Nsu EFTLNNPDAIGCLRMNMD--GNNLTVLVDPMHLQQVMWNLCNNAWRHSRQDENAITVLIR 451

Nfl EFTLNNPDAIGCLRMNMD--GNNLTVLVDPMHLQQVMWNLCNNAWRHSRQDENAITVLIR 451

Nci EFLLNNPDAVGCIRLDMQ--GNHLTAYFDSAHLRQIMWNLCNNAWRHSSKRSGSITVVVR 455

Npo EFLLGHPDAAGCIRPDIQ--GGSPTAYFDPAHLRQIMWNLANNAWRHSRKQPGSISVTIR 455

Nla EFLLNHPDAAGCIRPDIQ--GGSPTAYFDLAHLRQIMWNLANNAWRHSRKQPGSISVTIR 455

Nme EFLLGHPDAAGCIRLDMQ--GNHPTAYFDPAHLRQIMWNLANNAWRHSRKQPGSISVTIR 455

Pae EYPGRLR---NDSQLHLQLGAGDIQTRMDPHQLNQVLSNLVQNGLRYSAQAHGRGQVWLS 448

Kki EFQLTRPESAGCLRVDLP--KECLTAEFDIAHLQQIVWNLCNNAWRHSKKQRDSVVVSII 462

*: : : . * ::.*:: ** :*. ::. : : :

Nmus P--SG-RIHISIVVADNGTGVPPEVRNHLFEPFFTTDKQGTGLGLYVARELAHANLGQLH 510

AP206 P--SG-RMHISIVVADNGKGISPEVRNHLFEPFYTTEKQGTGLGLYVARELAHANMGQLH 511

Nwe A--SG-RMHVSILVMDSGMGVPPDIRTRLFEPFFTTEKRGTGLGLYVARELAHANMGQLH 510

Nsh P--LQDRHAVSLMVIDDGPGVPAENQAQLFEPFFTTRAEGTGLGLYVARELAHANLGQLD 514

Nwa P--SG-KMHISIVVADNGSGVAPEVRNRLFEPFFTTEKTGTGLGLYVARELAHANLGQLH 521

Nel P--SG-KLNVSIVVSDDGPGAAPEIRNQLFEPFFTTEKGGTGLGLYVARELAHANLGQLH 511

Nsi P--SG-RMHISIVVADNGKGISPEVRNHLFEPFYTTEKQGTGLGLYVARELAHANMGQLH 511

Nmu P--SG-RMHISIVVADNGKGISPEVRNHLFEPFYTTEKQGTGLGLYVARELAHANMGQLH 510

Nsu S--SG-RMHISIVVADNGKGVPPDVRNHLFEPFYTTEKQGTGLGLYVARELAHANMGQLH 508

Nfl P--SG-RMHISIVVADNGKGVPPDVRNHLFEPFYTTEKQGTGLGLYVARELAHANMGQLH 508

Nci P--AQ-KNTISILFADDGGGVPPEVREHLFEPFYTTAKNGTGLGLYVARELAHANFGDLT 512

Npo P--AQ-KNTVCILFADDGGGVPPEVQEHLFEPFYTTAENGTGLGLYVARELAHANFGDLT 512

Nla P--AQ-KNTVCILFADDGGGVPPEVQEHLFEPFYTTAENGTGLGLYVARELAHANFGDLT 512

Nme P--AQ-KNTVCILFADRPKCR-----NTCSNPFTPRRKTAPASGCMSPANWRT-PISAI- 505

Pae LARDPESDLPVLEVIDDGPGVPADKLNNLFEPFFTTESKGTGLGLYLSRELCESNQARID 508

Kki P--DGDT--IHLRVQDDGPGVAPKILEHLFEPFQTNQAEGTGLGLYVSHELAHANKGDLI 518

: . * :** . . * : . :

Nmus YHPEMNG---FEL--ILPKETDEQP- 530

AP206 YHPEMNG---FEL--ILPREHNE--- 529

Nwe YHPEMNG---FEL--ILPRDYNE--- 528

Nsh YHPAVKG---FEL--ILPRENDE--- 532

Nwa YHPEMNG---FEL--ILPRDESNDEA 542

Nel YHPELNG---FEL--ILPKDNNHEE- 531

Nsi YHPEMNG---FEL--ILPREHNE--- 529

Nmu YHPEMNG---FEL--ILPREHNE--- 528

Nsu YHPEMNG---FEL--ILPKEQAQDE- 528

Nfl YHPEMNG---FEL--ILPKEQAQDE- 528

Nci YLPEAKC---FEL--TLPEKSND--- 530

Npo YLPEAKC---FEL--TLPEKTND--- 530

Nla YLPEAKC---FEL--TLPEKSND--- 530

Nme -------------------------- 505

Pae YRNREEGGGCFRITFAHPRKLS---- 530

Kki YDGKNKA---FEL--IMPRKLS---- 535

**Fig. S1. Alignment of the deduced amino acid sequences of animal and human *Neisseria* Nps.** Residues boxed in blue comprise the predicted transmembrane domain. Residues boxed in orange form part of the phospho-acceptor and dimerization domains. Also included is the highly conserved Histidine (green box). Residues boxed in yellow comprise the putative phosphatase motif (ExxN). Residues boxed in purple correspond to the ATP-binding domain. Abbreviations: Nmus, *N. musculi*; AP206, *Neisseria species* isolated from a rhesus macaque (Weyand *et al.*, 2013); Nwe, *N. weaveri*; Nsh, N, *shayeganii*; Nwa, *N. wadsworthii*; Nel*, N. elongata*; Nmu, *N. mucosa*; Nsi, *N. sicca*; Nsu, *N. subflava*; Nfl, *N. flavescens*; Nci, *N. cinerea*; Npo, *N. polysaccharea*; Nla, *N. lactamica*; Nme, Nme, *N. meningitidis*; *N. gonorrhoeae*, Ngo; *Pseudomonas aeruginosa*, Pae; *Kingella kingae*, Kki; (†) highly conserved arginine in PilS important for interaction with the N-terminus of PilE; (*) identical residues; (:) highly conserved residues; and (.) semi-conserved residues.


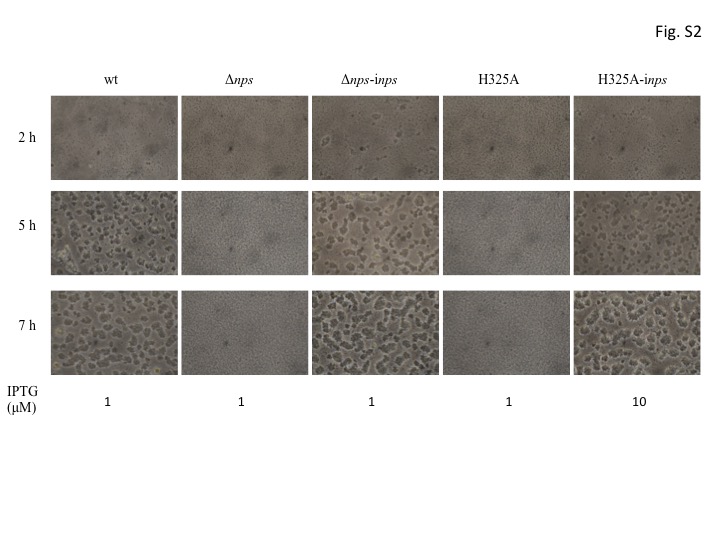


H325A+i*nps*

H325A

Δ*nps*+i*nps*

Δ*nps*

wt

**Fig. S2. Aggregation phenotype of bacteria grown in microtiter wells.** Nel 29315 wt, NelΔ*nps*, NelΔ*nps*+i*nps,* NelΔ*nps*H325A and*,* NelΔ*nps+*i*nps* were grown in GBC broth with Kellogg’s supplements and varying concentrations of IPTG. Bacteria were visualized at 2 h, 5 h and 7 h post-inoculation.


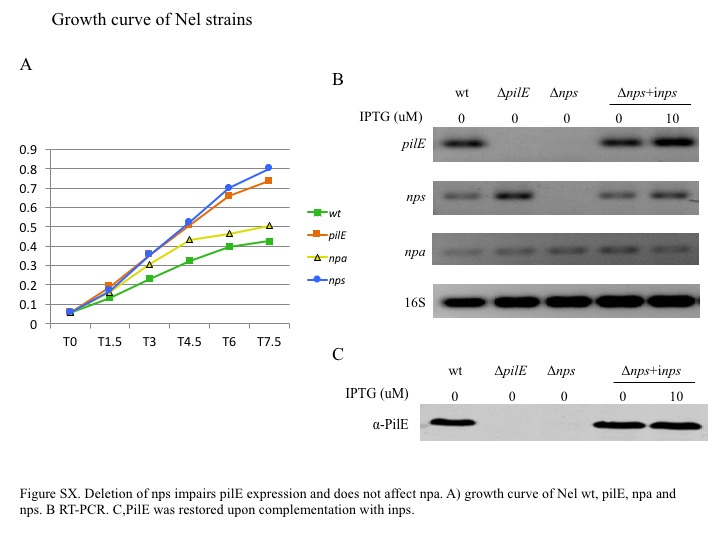


**Fig. S3. Deletion of *nps* impairs *pilE* expression and does not affect *npa***. A) Growth curve of Nel wt, Δ*pilE*, Δ*npa* and Δ*nps*. B) RT-PCR showing *pilE*, *nps*, and *npa* mRNA levels in Nel wt, Δ*nps* and Δ*nps*+*inps*; *npa* mRNA was not affected by the deletion of *nps* or complementation with the inducible construct (i*nps*). Controls are strain Nel Δ*pilE*, and a reaction to amplify 16S rRNA.


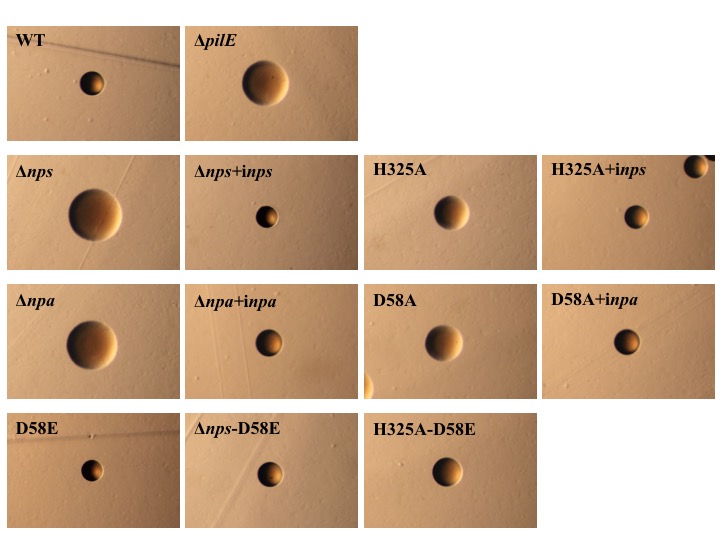


**Fig. S4. Colony morphology of *N. elongata* wild type and mutants.** Micrographs were acquired at 18X magnification using a Canon EOS 60D camera attached to a dissecting microscope.


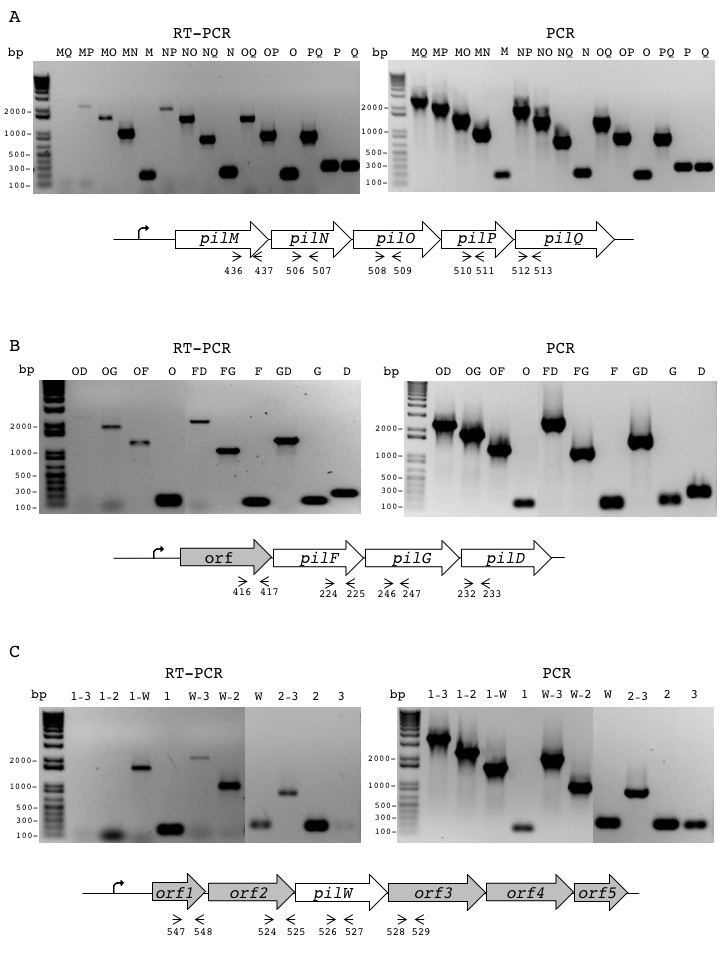


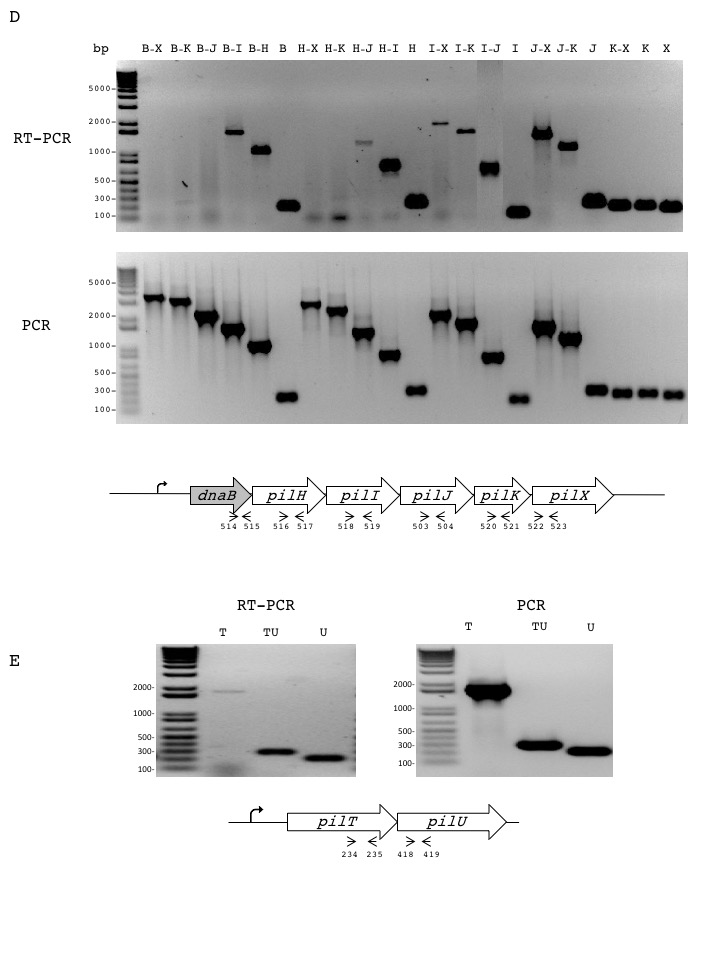


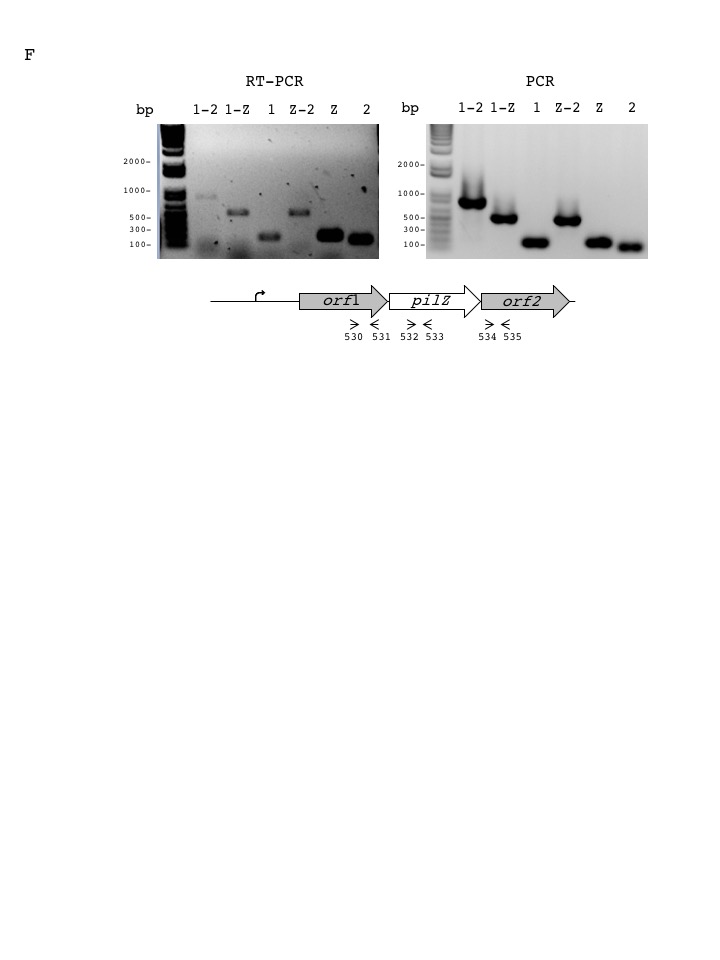


**Fig. S5. Organization of the Tfp biogenesis and function genes**. Tfp genes are organized in 10 different loci, some of them are cotranscribed as a single unit. An RT-PCR reaction was used to determine which genes are in an operon. A PCR reaction with DNA was used as control for the amplification. A) *pilM*, *pilN*, *pilO*, *pilP* and *pilQ*, B) *pilF*, *pilG* and *pilD* are forming an operon with an orf that encodes a putative inorganic phosphate transporter (NEIELOOT_00092); C) *pilW* (NEIELOOT_02585) is the third gene of a large operon, the rest of the genes are not known to be part of the Tfp, orf1 (NEIELOOT_02582), orf2 (NEIELOOT_02583), orf3 (NEIELOOT_02586), orf4 (NEIELOOT_02587) and orf5 (NEIELOOT_02588); D) *pilH*, *pilI*, *pilJ*, *pilK* and *pilX* are transcribed as a unit with an *dnaB* ; E) *pilT* and *pilU* are transcribed as an operon; F) *pilZ* is the middle gene of an operon together with orf1 (NEIELOOT_01544) and orf2 (NEIELOOT_01542). Not shown are *pilV*, *pilC*, *pilT2* and *pilE* since they do not belong to any operon. The graphs underneath each RT-PCR show the gene organization. The arrows correspond to each gene, grey arrows indicate genes that are not known to be involved in Tfp biogenesis or function. The primers used for the study are indicated with an arrowhead, numbers below the arrow corresponds to the primer used (see Table S2).

1 44

Nmus ---------MKAIQKGFTLI**E**LMIVIAIIGILAAIAL**P**MYQNYIARSQVARVMGESGNLK

AP206 ---------MYAQQKGFTLI**E**LLVVIAITAIMATIAL**P**NMSEWIASRRAASQAEQVANLL

Nwe ---------MKALQKGFTLI**E**LMIVIAIIGILAVIAL**P**AYQDYTARAQVSEAFSLAEGQK

Nsh MEKNIFSQKRVKISAGFTLV**E**MMIVLAIIGILAAIAL**P**SYSYYIERTNLATAKNELVELV

Nwa ---------MKAVQKGFTLI**E**LMIVIAIIGILAAIAL**P**MYQNYIARSQVTRAMGEAGNLK

Nel ---------MKAMQKGFTLI**E**LMIVIAIIGILAAIAL**P**AYQDYTVRSKVSEALIAASSPK

Nsi ---------MKAIQKGFTLI**E**LMIVVAIIGILAAIAL**P**MYGDYTARAQATEGYELLGGMK

Nmu ---------MKAIQKGFTLI**E**LMIVVAIIGILAAIAL**P**MYGDYTARAQATEGYELLGGMK

Nsf ---------MKAIQKGFTLI**E**LMIVIAIIGILAVIAL**P**AYQDYTARAQVSEAISLMEGQK

Nfl ---------MKAIQKGFTLI**E**LMIVIAILGILAVVAL**P**AYQDYTVRAKISEGLGLAEPAK

Nci ---------MKAIQKGFTLI**E**LMIVIAILGILAVIAF**P**AYQDYTIRAKVSEGLNLAAPAK

Npo ---------MKAIQKGFTLI**E**LMIVIAIVGILAAVAL**P**AYQDYTARAQMSEALTLAEGQK

Nla ---------MKAIQKGFTLI**E**LMIVIAIVGILAAVAL**P**AYQDYTARAQMSEALTLAEGQK

MC58 ---------MNTLQKGFTLI**E**LMIVIAIVGILAAVAL**P**AYQDYTARAQVSEAILLAEGQK

FAM18 ----------MTDNRGFTLV**E**LISVVLILSVLALIVY**P**SYRNYIEKAKINAVRAALLENA

MS11 ---------MNTLQKGFTLI**E**LMIVIAIVGILAAVAL**P**AYQDYTARAQVSEAILLAEGQK

Pae ----------MKAQKGFTLI**E**LMIVVAIIGILAAIAI**P**QYQNYVARSEGASALASVNPLK

**** *****:: .: : .::* :. ***** .

**Fig. S6. Alignment of the deduced amino acid sequences of animal and human *Neisseria* PilE.** Highly conserved E5 and P22 residues predicted to interact with Nps are bolded. Site of PilD cleavage site is underlined. Numbers above the aligned sequences indicate amino acid position. Abbreviations: Nmus, *N. musculi*; AP206, *Neisseria species* isolated from a rhesus macaque (Weyand *et al.*, 2013); Nwe, *N. weaveri*; Nsh, N, *shayeganii*; Nwa, *N. wadsworthii*; Nel*, N. elongata*; Nmu, *N. mucosa*; Nsi, *N. sicca*; Nsu, *N. subflava*; Nfl, *N. flavescens*; Nci, *N. cinerea*; Npo, *N. polysaccharea*; Nla, *N. lactamica*; Nme, Nme, *N. meningitidis*; *N. gonorrhoeae*, Ngo; *Pseudomonas aeruginosa*, Pae. (*) identical residues; (:) highly conserved residues; and (.) semi-conserved residues.

**Fig. S7. Construction of the *nps* deletion mutant**. The *nps* and *npa* ORFs overlap by 11 bases. An in-frame deletion of *nps* was achieved by removing 92% of the 5' end of the *nps* ORF and inserting a kanamycin (Km) resistance gene from plasmid pHSS6 (Seifert *et al.*, 1986) at this site. The Km gene was then removed by transforming Nel Δ*nps* Km^R^ with a 321 bp fragment of DNA containing the remaining117 bases of *nps*. The transformation mix was serially diluted and plated on GCB agar without antibiotics. After 24 h of incubation, individual colonies were picked onto GCB agar, and colonies from this plate were replica-plated on GCB-Km agar. Km-sensitive colonies were analyzed by PCR and sequenced to confirm deletion of the Km gene and fusion of *nps/npa*.


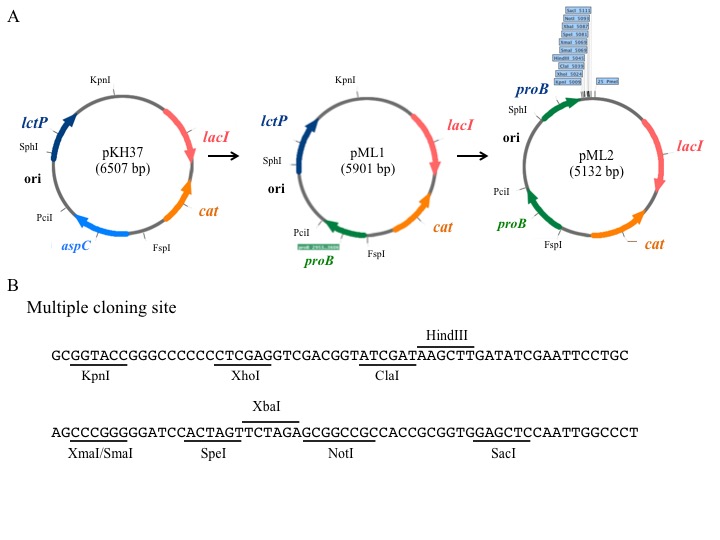


**Fig. S8. *N. elongata* complementation plasmid pML2.** A, Diagram showing construction of pML2, which was used for complementation experiments in *N. elongata.* Briefly, plasmid pKH37 was modified by replacing the *lctP* and *aspC* sites with *proB*. B, Multiple cloning site sequence in pML2.


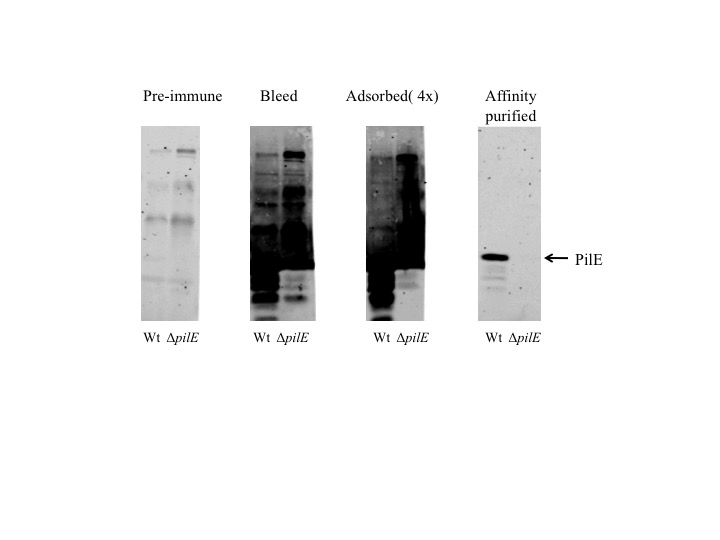


**Fig. S9. Reactivity of polyclonal α-PilE_Nel_ antibodies**. Western blot showing the purity of the polyclonal α-PilE antibodies generated in rabbits (Alpha Diagnostic Intl. Inc). Antibodies specific to PilE were purified from the final bleed by adsorption with Nel Δ*pilE* cells, followed by affinity purification using purified Nel PilE (see methods).

Table S1. List of strains and plasmids used in this study.

| Strain | Genotype or relevant characteristics | Source |
| --- | --- | --- |
| ***N. elongata*** | |  |
| 29315 | *N. elongata* subspecies glycolytica wild type, P+ | (Marri *et al.*, 2010) |
| NelΔ*pilE* | Nel 29315Δ*pilE* Km^R^ | (Higashi *et al.*, 2011) |
| NelΔ*nps* | Nel 29315Δ*nps* | This study* |
| NelΔ*nps+inps* | Nel 29315Δ*nps* carrying inducible *nps* under *lac* promoter, Cm^R^ | This study |
| Nel *nps* H325A | Nel 29315 *nps* carrying a point mutation where histidine 325 was replaced by alanine, Km^R^ | This study |
| Nel *nps* H325A+inps | Nel *nps* H325A carrying inducible *nps* under lac promoter, Cm^R^ | This study |
| NelΔ*npa* | Nel 29315Δ*npa* Km^R^ | (Rendón *et al.*, 2013) |
| NelΔ*npa*+inpa | Nel 29315Δ*npa* carrying inducible *npa* under *lac* promoter, Km^R^ and Cm^R^ | This study |
| Nel *npa* D58A | Nel 29315 *npa* carrying a point mutation where aspartate 58 was replaced by alanine, Km^R^ | This study |
| Nel *npa* D58A-i*npa* | Nel *npa* D58A carrying inducible *npa* under *lac* promoter, Km^R^ and Cm^R^ | This study |
| Nel *npa* D58E | Nel 29315 *npa* carrying a point mutation where aspartate 58 was replaced by glutamate, Km^R^ | This study |
| Nel Δ*nps-npa* D58E | Nel 29315Δ*nps npa* carrying a point mutation where aspartate 58 was replaced by glutamate, Km^R^ | This study |
| Nel *nps* H325A-*npa* D58E | Nel *nps* H325A with *npa* carrying a point mutation where aspartate 58 was replaced by glutamate, Km^R^ | This study |
|  |  |  |
|  |  |  |
|  |  |  |
| ***E. coli*** |  |  |
| DH5α | f*huA*2 *lac*(del)U169 *phoA gln*V44 Φ80' *lacZ*(del)M15 *gyrA*96 *recA*1 *relA*1 *endA*1 *thi*-1 *hsdR*17 | Lab collection |
|  |  |  |
| **Plasmids** |  |  |
| pKH37 | Amp^R^, Cm^R^ | (Kohler *et al.*, 2007) |
| pML2 | Modified pKH37 to be used in Nel, Amp^R^, Cm^R^ | This study* |
| pML2-inps | pML2 carrying *nps* under an inducible *lac* promoter | This study |
| pML2-inpa | pML2 carrying *nps* under an inducible *lac* promoter | This study |
| pKm-*nps*-*npa* | pUC19 carrying a Km cassette and *nps*-*npa*, used to introduce point mutations. | This study* |
| pHSS6 | Source of Km cassette used to make mutants | (Seifert *et al.*, 1986) |
|  |  |  |

*See methods for description on how it was constructed

Table. S2. Primers used in this study

| Primer | Sequence | Target | Reference |  |  |  |
| --- | --- | --- | --- | --- | --- | --- |
| RT-PCR |  |  |  |  |  |  |
| MR44 | aggtttcaccctgatcgagtt | F- *pilE*_Nel_ | (Rendón *et al.*, 2013) |  |  |  |
| MR45 | tcggaaactttagaacgtacagtg | R- *pilE*_Nel_ | (Rendón *et al.*, 2013) |  |  |  |
| MR209 | cctgaccgacagcctagaag | F- *rpoN*_Nel_ | (Rendón *et al.*, 2013) |  |  |  |
| MR210 | atgatgtcggcggtgtagtc | R- *rpoN*_Nel_ | (Rendón *et al.*, 2013) |  |  |  |
| MR248 | gccttcgggttgtaaaggac | F- 16S | (Rendón *et al.*, 2013) |  |  |  |
| MR249 | gattaacgctcgcaccctac | R- 16S | (Rendón *et al.*, 2013) |  |  |  |
| MR295 | TGCGGTTTATGCCTCTTCTC | F- *parC* | This work |  |  |  |
| MR297 | CGGTTTCGACGATAAATTCC | R- *parC* | This work |  |  |  |
| MR299 | TGAGCGACCAATTGAGTTTG | F- *nps* | This work |  |  |  |
| MR300 | TCCGGATCAATCACAATCAC | R- *nps* | This work |  |  |  |
| MR298 | TCGGGTTTGGAAGTGGTG | F- *npa* | This work |  |  |  |
| MR296 | CTTCGGGCTCGTTTACTTTG | R- *npa* | This work |  |  |  |
| MR436 | GCAAGGGGAGAAAATTGTTG | F-*pilM* | This work |  |  |  |
| MR437 | TTTTGCATTACTTGCGCTTG | R-*pilM* | This work |  |  |  |
| MR506 | taaacgtttcgaaggtgctc | F-*pilN* | This work |  |  |  |
| MR507 | gctcaggctgtccaaatacc | R-*pilN* | This work |  |  |  |
| MR508 | AGAGTTGGAGGCACTTCGAG | F-*pilO* | This work |  |  |  |
| MR509 | CCGTCATTTATTGGCTCCAG | R-*pilO* | This work |  |  |  |
| MR510 | acccctcctatgcaaccaac | F-*pilP* | This work |  |  |  |
| MR511 | tgattcgcccatagttttgac | R-*pilP* | This work |  |  |  |
| MR512 | GGACCTTCTTCTCGTGTTGC | F-*pilQ* | This work |  |  |  |
| MR513 | TGAGCACTGGGTGTCGTATG | R-*pilQ* | This work |  |  |  |
| MR416 | AATAGGTGTTTCCGCACCTG | F-orf (NEIELOOT_00092) | This work |  |  |  |
| MR417 | CGCACCAACCAAGATATGTG | R-orf (NEIELOOT_00092) | This work |  |  |  |
| MR224 | GGTGAGATTCGTGATTTGGAAAC | F-*pilF* | This work |  |  |  |
| MR225 | AATGGTGCGACACCCATATT | R-*pilF* | This work |  |  |  |
| MR246 | cagagggcatgtgaatcctt | F-*pilG* | This work |  |  |  |
| MR247 | agtacaccaccagcctcacc | R-*pilG* | This work |  |  |  |
| MR232 | ATGATGGAACGTGAATGGAC | F- *pilD* | This work |  |  |  |
| MR233 | GCCGAATACACAAGCAGTCA | R- *pilD* | This work |  |  |  |
| MR547 | GAAGGTGAGGACGCAGTAGC | F-orf1(NEIELOOT_02582) | This work |  |  |  |
| MR548 | AGCAGCATTTTCCAAGCTGT | R-orf1(NEIELOOT_02582 | This work |  |  |  |
| MR524 | TCGCCGTTATTTGGTTAAGG | F-orf2(NEIELOOT_02583) | This work |  |  |  |
| MR525 | GCAAAATATCGCGGAAAATC | R-orf2(NEIELOOT_02583) | This work |  |  |  |
| MR526 | TACGCCTAAAACCCGATAGC | F-*pilW* | This work |  |  |  |
| MR527 | CGTTCCAAATATGCTTGTGC | R-*pilW* | This work |  |  |  |
| MR528 | GGAATTTGGCCGGATATTAAG | F-orf3(NEIELOOT_02586) | This work |  |  |  |
| MR529 | ATTGAAACGGCTTGCTCATC | R-orf3(NEIELOOT_02586) | This work |  |  |  |
| MR514 | CCCGCTCATTAAAATCCTTG | F-*dnaB* | This work |  |  |  |
| MR515 | AGCCAAACCTTTCATTGGTG | R-*dnaB* | This work |  |  |  |
| MR516 | CAAAAATGTGGGATTTGATGG | F-*pilH* | This work |  |  |  |
| MR517 | CTAACTTGCCCGCCTGATAC | R-*pilH* | This work |  |  |  |
| MR518 | ATGCCGAATTAAATGCAAGC | F-*pilI* | This work |  |  |  |
| MR519 | CCCCAATCTGAAACATCACC | R-*pilI* | This work |  |  |  |
| MR503 | CGGCTACGAGTGATCAGACA | F-*pilJ* | This work |  |  |  |
| MR504 | TTTGCACCACTGGACGAATA | R-*pilJ* | This work |  |  |  |
| MR520 | ATCGCAAGATGACAATGCAG | F-*pilK* | This work |  |  |  |
| MR521 | AGGCTTTTGCTTCCTCCTTC | R-*pilK* | This work |  |  |  |
| MR522 | GCAGCTTGGTTTTACCTTGG | F-*pilX* | This work |  |  |  |
| MR523 | CCGTTTTAATAACATCCGCAAC | R-*pilX* | This work |  |  |  |
| MR545 | cttacgcggcttcactttg | F-*pilV* | This work |  |  |  |
| MR546 | tctaatcgctgtgcattgttc | R-*pilV* | This work |  |  |  |
| MR252 | GGGCGAGTTGGTCTGTGTAG | F-*pilC* | This work |  |  |  |
| MR253 | GCTTGGAGTATCACCTGCTTG | R-*pilC* | This work |  |  |  |
| MR234 | AATTACCGACTTACTCGCGTTC | F-*pilT* | This work |  |  |  |
| MR235 | GCTCGGAATGGTACGGAATAC | R-*pilT* | This work |  |  |  |
| MR418 | tcgaagacccgatcgaatac | F-*pilU* | This work |  |  |  |
| MR419 | ggcgtggatggtgaagatac | R-*pilU* | This work |  |  |  |
| MR236 | ATTTTCGTCGGCGGTACA | F-*pilT2* | This work |  |  |  |
| MR237 | TTCGCGTTGGGTAACGATG | R-*pilT2* | This work |  |  |  |
| MR530 | cggattggcacaacaaaac | F-orf1 (NEIELOOT_01544) | This work |  |  |  |
| MR531 | attcggtgagcaggtattcg | R-orf1(NEIELOOT_01544) | This work |  |  |  |
| MR532 | acatgccctttctcgaacac | F-*pilZ* | This work |  |  |  |
| MR533 | gatttcgtctttgccgaatg | R-*pilZ* | This work |  |  |  |
| MR534 | actgccacctcaacttcgac | F-orf2 (NEIELOOT_01542) | This work |  |  |  |
| MR535 | tagatgtgcgcgtgttgttc | R-orf2 (NEIELOOT_01542) | This work |  |  |  |
|  |  |  |  |  |  |  |
| Plasmids pML2, pML2-i*nps*, pML2-i*npa* | | | |  |  |  |
| MR86 | aggatcGAGATTTTCAGGAGCTAAGGAAG | F- Cm -BamHI | This work |  |  |  |
| MR87 | gagctcCTGCCACTCATCGCAGTA | R- Cm -SacI | This work |  |  |  |
| MR346 | Atcgtttctcgagttgtctggcggtatttctgg | F-*proB* 5’ - FspI | This work |  |  |  |
| MR347 | AtcgtttacatgtTTGGCTTGCGGATTTTTG | R-*proB* 5’ -PciI | This work |  |  |  |
| MR348 | AtcgtttgcatgcACCGGCGGCATGTACACC | F-*proB* 3’ - SphI | This work |  |  |  |
| MR349 | Atcgtttggtaccgctgtcaaacggcaaagg | R-*proB* 3’- KpnI | This work |  |  |  |
| MR350 | atcgtttTTAATTAAggagtacgaatATGGATAAATCTTTTTTCC | F-*nps* -PacI | This work |  |  |  |
| MR351 | atcgtttgagctcCAGGGTCATTTCCATCAGGT | R-*nps* -SacI | This work |  |  |  |
| MR216a | atcgtttTTAATTAAggagtacgaatATGAAGAGTAACGATCTGCAA | F-*npa* -PacI | This work |  |  |  |
| MR217a | atcgtttgagctctCTACCAACGCTTCCAGGTT | R-*npa -*SacI | This work |  |  |  |
|  |  |  |  |  |  |  |
| To construct the plasmid to introduce point mutations in *nps* or *npa* | | | |  |  | This work |
| MR333 | atcgttt*gttaac*aagagaaagcaggtagcttgcag | F-Km - HincII | This work |  |  |  |
| MR334 | atcgtttaagcttAACAGGCCGTCTGAAAAACCTTTTTCAGACGGCCTGTTTgctcagaagaactcgtcaagaagg | R-Km - HindIII | This work |  |  |  |
| MR313b | atcgttt*GAATTC*GGGAAGGTTTTGGACGTATC | F-*nps*-*npa* - EcoRI | This work |  |  |  |
| MR314 | atcgttt*GGATCC*aatcatccgtgaaaccatagac | R-*nps*-*npa*- BamHI | This work |  |  |  |
| MR356 | tcgctggtattgacgGAAatgcgtatgcccgacggt | F-*npa*D58E | This work |  |  |  |
| MR357 | ATAGTCGTTGTTTTCCAATTTTTCTTTGGC | R-*npa* D58A and D58E | This work |  |  |  |
| MR398 | tcgctggtattgacgGCCatgcgtatgcccgacgg*t* | F-npa D58A | This work |  |  |  |
| MR388 | accgccaatctggcgGCCgaaatccgcaacccg | F-nps H325A | This work |  |  |  |
| MR384 | CAGCTGGCCGAGGGAAGCCAGTTTGGTTGC | R-nps H325A | This work |  |  |  |
|  |  |  |  |  |  |  |
|  |  |  |  |  |  |  |
|  |  |  |  |  |  |  |
| Mapping transcriptional start site (5’ RACE) | | | |  |  |  |
| MR358 | GATAAACCATTTTGATGGCATTCTG | *pilMNOPQ* | This work |  |  |  |
| MR544 | ACCTGCTTGGCGCGAAACGGTTTTT | *pilW* | This work |  |  |  |
| MR354 | ATGTAGCGCAGCCAGTTGAAG | *pilT*2 | This work |  |  |  |
| MR542 | CGTTGCTTGTTCGCACAGCAGTGTAG | *pilZ* | This work |  |  |  |

Table. S3. Transcription starting sites of Nel genes involved in Tfp assembly and retraction

| **Gene/operon** | | **Sequence*** |  |
| --- | --- | --- | --- |
| ***pilMNOPQ*** | ttacccaagaaggcattaaataaagtaaaatgggtttaacgt***t***taga...85...ATG | |  |
| ***pilFGD*** | ND | |  |
| ***pilW*** | acaaataaggcggaatacggaaaaacttgttacaatgcgccg***g***catt...47...ATG | |  |
| ***pilHIJKX*** | ND | |  |
| ***pilV*** | ND | |  |
| ***pilC*** | ND | |  |
| ***pilTU*** | ND | |  |
| ***pilT*2** | aaaccgcctaccgaccgcgcaaatcacagtataatcgccat***c***tttca...60...ATG | |  |
| ***pilZ*** | ggccactgttgcaaaaacgggcgcggctatataatgcgccc***g***tccga...17...ATG | |  |

*underlined sequences correspond to the putative -35 and -10 regions. TIS is bolded and italicized.

ND not done

SReferences

Higashi, D.L., N. Biais, N.J. Weyand, A. Agellon, J.L. Sisko, L.M. Brown & M. So, (2011) *N. elongata* produces type IV pili that mediate interspecies gene transfer with *N. gonorrhoeae*. *PLoS One* **6**: e21373.

Kohler, P.L., H.L. Hamilton, K. Cloud-Hansen & J.P. Dillard, (2007) AtlA functions as a peptidoglycan lytic transglycosylase in the *Neisseria gonorrhoeae* type IV secretion system. *J Bacteriol* **189**: 5421-5428.

Marri, P.R., M. Paniscus, N.J. Weyand, M.A. Rendón, C.M. Calton, D.R. Hernandez, D.L. Higashi, E. Sodergren, G.M. Weinstock, S.D. Rounsley & M. So, (2010) Genome sequencing reveals widespread virulence gene exchange among human *Neisseria* species. *PLoS One* **5**: e11835.

Rendón, M.A., A.M. Hockenberry, S.A. McManus & M. So, (2013) Sigma factor RpoN (sigma54) regulates *pilE* transcription in commensal *Neisseria elongata*. *Mol Microbiol* **90**: 103-113.

Seifert, H.S., E.Y. Chen, M. So & F. Heffron, (1986) Shuttle mutagenesis: a method of transposon mutagenesis for *Saccharomyces cerevisiae*. *Proc Natl Acad Sci U S A* **83**: 735-739.

Weyand, N.J., A.M. Wertheimer, T.R. Hobbs, J.L. Sisko, N.A. Taku, L.D. Gregston, S. Clary, D.L. Higashi, N. Biais, L.M. Brown, S.L. Planer, A.W. Legasse, M.K. Axthelm, S.W. Wong & M. So, (2013) *Neisseria* infection of rhesus macaques as a model to study colonization, transmission, persistence, and horizontal gene transfer. *Proc Natl Acad Sci U S A* **110**: 3059-3064.
